# A low molecular weight multifunctional theranostic molecule for the treatment of prostate cancer

**DOI:** 10.7150/thno.68715

**Published:** 2022-02-21

**Authors:** Xinning Wang, Rongcan Sun, Jing Wang, Jing Li, Ethan Walker, Aditi Shirke, Gopolakrishnan Ramamurthy, Lingpeng Shan, Dong Luo, Lauren Carmon, James P. Basilion

**Affiliations:** 1 Department of Biomedical Engineering, Case Western Reserve University, 11100 Euclid Ave, Wearn Building B-49, Cleveland, OH, USA, 44106.; 2 Department of Radiology, 11100 Euclid Ave, Wearn Building B-44, Case Western Reserve University, Cleveland, OH, USA, 44106.; 3 Department of Chemistry, Case Western Reserve University, 2080 Adelbert Rd, Cleveland, OH, USA, 44106.; 4 Department of Population and Quantitative Health Sciences, Case Western Reserve University, 2103 Cornell Rd, Cleveland, OH, USA, 44106.; 5 Department of Nutritional Biochemistry and Metabolism, 2109 Adelbert Rd, Cleveland, OH, USA, 44106.

**Keywords:** prostate cancer, PSMA, MMAE, IR700, multifunctional molecule

## Abstract

**Rationale:** Although surgery and radiation therapy in patients with low risk prostate cancer appear appropriate and effective, those with high-risk localized disease almost always become hormone refractory and then rapidly progress. A new treatment strategy is urgently needed for patients with high-risk localized prostate cancer, particularly an approach that combines two drugs with different mechanisms. Combinations of photodynamic therapy (PDT) and chemotherapy have shown synergistic effects in clinical trials, but are limited by off-target toxicity. Prostate specific membrane antigen (PSMA) is a well-established biomarker for prostate cancer. Here we describe the use of a PSMA ligand to selectively and simultaneously deliver a potent microtubule inhibiting agent, monomethyl auristatin E (MMAE), and a PDT agent, IR700, to prostate cancers.

**Methods:** Using a bifunctional PSMA ligand PSMA-1-Cys-C6-Lys, we created a novel theranostic molecule PSMA-1-MMAE-IR700. The molecule was tested *in vitro* and *in vivo* for selectivity and antitumor activity studies.

**Results:** PSMA-1-MMAE-IR700 showed selective and specific uptake in PSMA-positive PC3pip cells, but not in PSMA-negative PC3flu cells both *in vitro* and* in vivo*. In *in vitro* cytotoxicity studies, when exposed to 690 nm light, PSMA-1-MMAE-IR700 demonstrated a synergistic effect leading to greater cytotoxicity for PC3pip cells when compared to PSMA-1-IR700 with light irradiation or PSMA-1-MMAE-IR700 without light irradiation. *In vivo* antitumor activity studies further showed that PSMA-1-MMAE-IR700 with light irradiation significantly inhibited PC3pip tumor growth and prolonged survival time as compared to mice receiving an equimolar amount of PSMA-1-IR700 with light irradiation or PSMA-1-IR700-MMAE without light irradiation.

**Conclusion:** We have synthesized a new multifunctional theranostic molecule that combines imaging, chemotherapy, and PDT for therapy against PSMA-expressing cancer tissues. This work may provide a new treatment option for advanced prostate cancer.

## Introduction

In the United States prostate cancer is the most common malignancy and the second leading cause of cancer death in men 1. It is estimated that 248,530 men will be diagnosed with prostate cancer and 34,130 men will die from the disease in 2021 1. Screening with serum prostate specific antigen (PSA) allows for 78% of prostate cancers to be diagnosed at the early localized stage, facilitating therapy with radical prostatectomy or radiation therapy 2. However, the clinical reality is that many men present with high-risk localized prostate cancer. High-risk localized prostate cancer accounts for 15% of all prostate cancer diagnosis and the rate has increased in recent years 3. These patients with high-grade tumors (local tumor stage T2c, Gleason score >7, and PSA level >20 µg/L) 4 have a high risk of biochemical recurrence resulting in a 5-year recurrence rate of approximately 70% 5. Furthermore, these patients have a significantly higher risk of metastatic disease and prostate cancer related mortality 6-9. Traditional single modality regimens for treating patients with high-risk localized diseases such as radical prostatectomy and radiation therapy have resulted in poor treatment responses, high failure rates and a high risk for both clinical and biochemical progressions (>50% at 5 years). Androgen deprivation therapy (ADT) with radiation therapy is now standard of care for patients with high-risk prostate cancer. However, the optimal duration of ADT remains undefined 3 and ADT is associated with multiple and significant side effects such as hot flashes, sexual dysfunction and skeletal mobility; almost all patients will develop resistance to ADT 10. Neoadjuvant ADT before radical prostatectomy has been tested in clinical trials with lack of improvement in survival for all studies 11-14. The role of ADT adjuvant therapy after radical prostatectomy is uncertain. Recently, Docetaxel-based adjuvant therapy has demonstrated modest improvements in treatment outcomes for high-risk localized disease, with overall survival benefits reported in one of the studies 15-17. However, the use of docetaxel is restricted by its toxicity (mostly neutropenia and neurotoxicity) and complex formulation (use of Cremophor^EL^) 18. In addition, all patients will ultimately develop resistance 19, 20. Consensus regarding optimal treatment of patients with high-risk localized prostate cancer has not been established. There is an unmet clinical need to develop new and potent molecularly targeted therapies to improve outcomes for men with high-risk localized prostate cancer, particularly a methodology that considers the use of a multimodal approach and that includes both local and systemic therapies.

Photodynamic therapy (PDT) is a light-based minimally-invasive therapy used clinically in the treatment of cancers and other diseases 21-24. During PDT, the non-toxic photosensitizer will be activated by specific light in the presence of oxygen and transfer energy to molecular oxygen resulting in the generation of reactive oxygen species (ROS) 25-27. Besides direct cell killing by ROS, PDT can damage tumor vasculature causing cancer death. PDT can also initiate a post-treatment immune response directed against tumor cells 25-27. Based on these mechanisms, PDT has the ability to bypass the several resistance mechanisms displayed by malignant cells 28-30. Due to the unique mechanisms of PDT and few side effects, it can be utilized in combination with chemotherapy to overcome chemo-resistant cancer and achieve a synergistic therapeutic effect with chemotherapy 28, 31-35. Such combination treatments have been tested clinically and demonstrated an enhanced anti-tumor response compared to either PDT or chemotherapy alone 35-38. Combination of PDT with chemotherapy has also been reported to improve treatment for late stage advanced lung cancer and nonresectable cholangiocarcinoma 39, 40.

Although intriguing results have been found for the treatment of cancer by combination of PDT and chemotherapy, the inherent drawbacks of conventional PDT and chemotherapy are not eliminated. Both are limited by off-target tissue accumulation leading to cell death in normal tissue. Another problem with current clinical protocols of combination treatment of PDT and chemotherapy is that each drug is administered separately and may not reach the tumor at the same time, so combined efficacy may be lost or muted. To obtain a better combined chemo-photodynamic therapeutic effect, a desirable drug delivery method that can simultaneously and selectively deliver anticancer drugs and photosensitizers to cancer cells is required.

**Figure 1:**
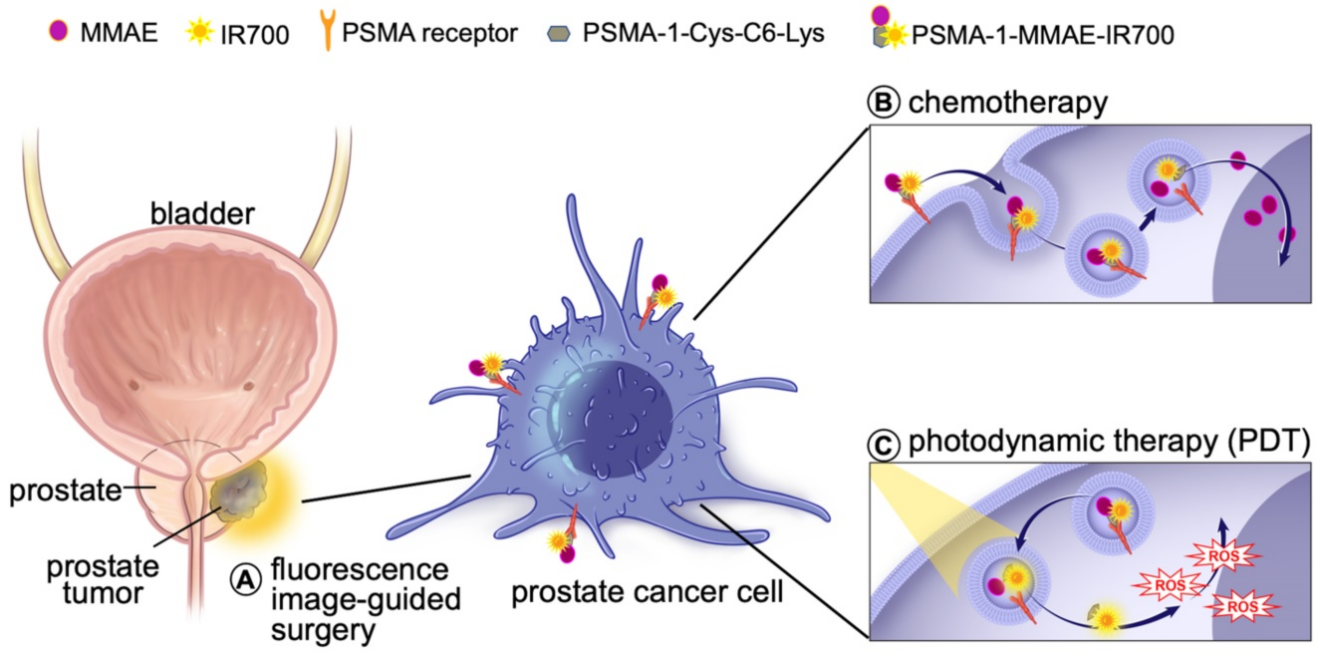
** Schematic illustration of mechnaism of the multifunctional theranostic molecule PSMA-1-MMAE-IR700.** After administration, PSMA-1-MMAE-IR700 will selectivly bind to PSMA receptors on prostate cancer cells and enter cancer cells through receptor mediated endocytosis. The fluorescence signal emitted by IR700 can be used for diagnosis and image-guided surgery of prostate cancer by detecting residual cancer (A). Internalized PSMA-1-MMAE-IR700 will be delivered to lysosomes, the conjugate will be digested by cathepsins to generate free MMAE and PSMA-1-IR700. Delivered as a prodrug 60, protease released MMAE will exert its chemotherapeutic effect (B), while PSMA-1-IR700 will generated reactive oxygen species and deploy photodynamic therapy when illuminated by 690 nm light 58 (C).

**Figure 2:**
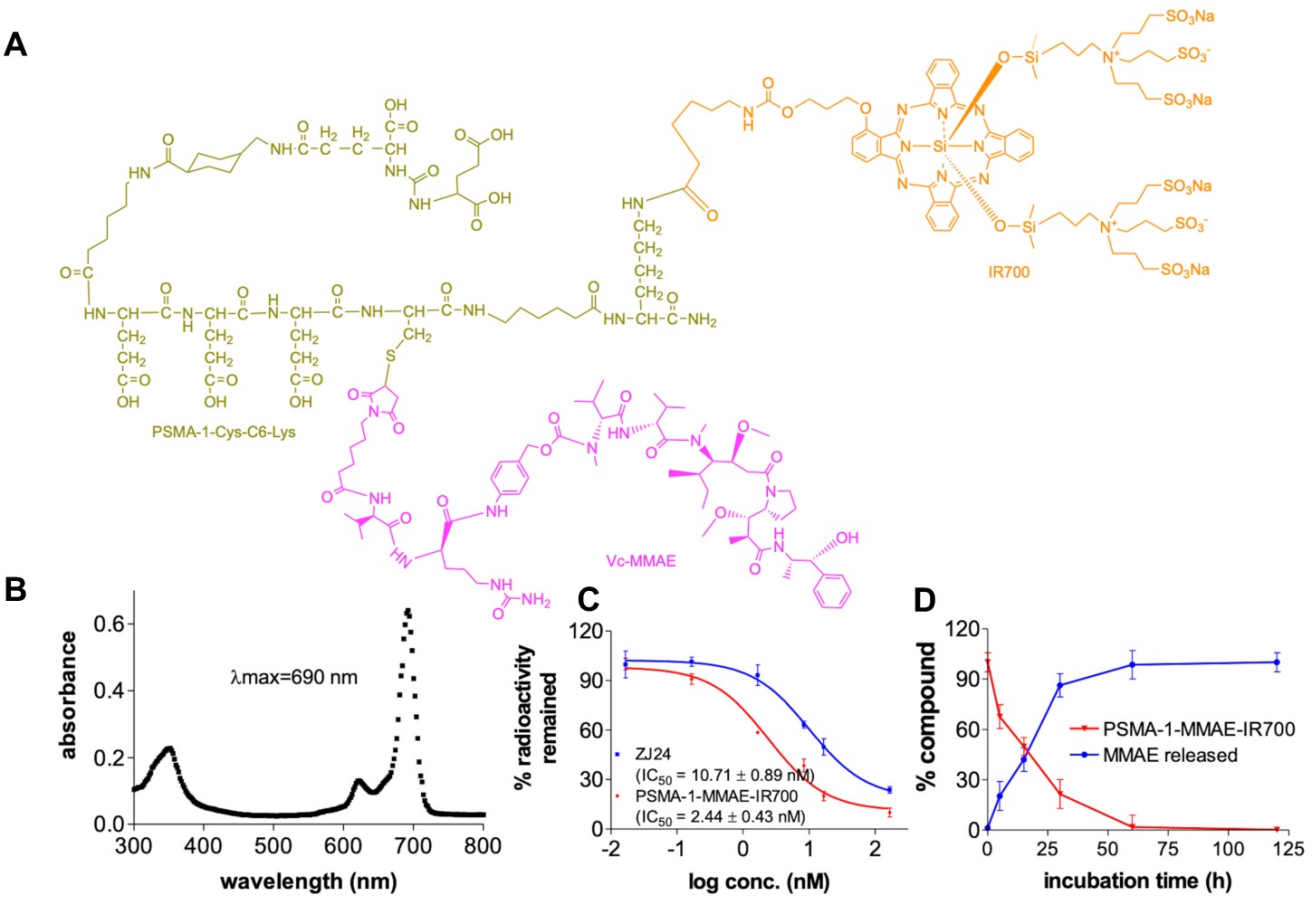
** Characterization of PSMA-1-MMAE-IR700. (A)** Structure of PSMA-1-MMAE-IR700. **(B)** Absorbance spectrum of PSMA-1-MMAE-IR700. **(C)** *In vitro* competition binding results of PSMA-1-MMAE-IR700. Values are mean ± SD of triplicates. **(D)** *In vitro* cathepsin cleavage of PSMA-1-MMAE-IR700. Values are mean ± SD of triplicates.

Prostate specific membrane antigen (PSMA) is a well-known biomarker for prostate cancer 41-46. Over the past decade, tremendous progress has been made with PSMA-targeted imaging agents 47-52 and radiotherapies 52-56. ^68^Ga-PSMA11 and ^18^F-labeled Pylarify have been approved by Food and Drug Administration (FDA) for positron emission tomography (PET) imaging of prostate cancer. We have successfully developed a high affinity PSMA ligand (PSMA-1) 57, and have utilized it to develop PSMA-targeted PDT agents 58, 59. Although our PSMA-targeted PDT conjugates showed effective tumor inhibition, some cancer cells were found to survive and tumor recurrences were eventually seen in immunocompromised mouse models after PDT treatment 58. Others have also reported that PDT is effective in small tumors but less effective for treating large tumors due to limited light penetration 35. Thus, it is necessary to develop a new method to enhance the effectiveness of PDT treatment. Combination of PDT with chemotherapy can provide a second treatment to kill remaining cancer cells that survive PDT and has been shown to be more effective than either treatment alone 35-38. Recently, we reported a PSMA-targeted chemotherapeutic prodrug PSMA-1-VcMMAE for the treatment of prostate cancer, which was effective but not decisive against prostate cancer 60. It is hypothesized that combination of PSMA-targeted PDT and PSMA-targeted MMAE will improve the antitumor efficacy. The objective of this study was to develop a multifunctional theranostic approach that combines a cytotoxic drug (MMAE), a photosensitizer (IR700) and a low molecular weight PSMA targeting ligand (PSMA-1-Cys-C6-Lys) into a single molecule, PSMA-1-MMAE-IR700, selectively and simultaneously delivering both chemotherapeutic drugs and photosensitizers to cancer cells (**Figures 1 and 2A**). In addition, IR700 emits near infrared light at around 700 nm with a fluorescence quantum yield at 0.24 61, therefore, this new approach can also be used for near infrared fluorescence detection of cancers (**Figure 1**). Our results showed that PSMA-1-MMAE-IR700 can be selectively delivered to PSMA-expressing cancer cells. More importantly, the PSMA-targeted covalent combination of PDT and chemotherapy agents showed significantly improved antitumor activity compared to individually PSMA-targeted treatment with PDT or chemotherapy, or the non-covalent simultaneous addition of both PSMA-targeted PDT and free MMAE.

## Methods

### Materials

PSMA-1-Cys-C6-Lys (Glu-CO-Glu'-Amc-Ahx-Glu-Glu-Glu-Cys-C6-Lys) was synthesized manually by solid phase peptide synthesis method as reported previously 57, 58, 60. VcMMAE was purchased from Creative Biolabs (Shirley, NY). IRDye700 NHS ester was purchased from Li-Cor Biosciences (Lincoln, NE). PSMA-1-IR700 was synthesized as previously reported 58. (S)-2-(3-((S)-5-amino-1-carboxypentyl)ureido)pentanedioic acid (ZJ24) was custom made by Bachem Bioscience Inc (Torrance, CA). Tritium labeled ZJ24 (N-[N-[(*S*)-1,3-dicarboxypropyl]carbamoyl]-*S*-[^3^H]-methyl-L-cysteine, ^3^H-ZJ24) was custom synthesized by GE Healthcare Life Sciences (Chicago, IL). All the other chemicals were purchased from Sigma-Aldrich (St. Louis, MO).

### High performance liquid chromatography (HPLC)

HPLC was performed on a Shimadzu HPLC system equipped with a SPD-20A prominence UV/visible detector and monitored at 220 nm and 254 nm 57, 58, 60. Semi-preparative HPLC was achieved using Luna 5m C18(2) 100 Å column (250mm × 10mm × 5 mm; Phenomenex) at a flow rate of 2.5 mL/min. Analytical HPLC was performed using an analytical Luna 5m C18(2) 100 Å column (250 mm × 4.6 mm × 5 mm; Phenomenex) at a flow rate of 0.8 mL/min. The gradient used to purify PSMA-1-MMAE-IR700 was 10% to 90% acetonitrile against 25 mM triethylammonium acetate (TEAA, pH 7.5) over 30 min.

### Synthesis of PSMA-1-MMAE-IR700 (Figure S1)

PSMA-1-VcMMAE was synthesized as previously reported 60. Briefly, PSMA-1-Cys-C6-Lys (2.6 mg, 2 μmol) (**Figure S2**) was dissolved in phosphate buffered saline (PBS), the pH of the solution was adjusted to 7.5 - 8.0, then Vc-MMAE (3.0 mg, 2.2 μmol) (BOC Sci.) in 500 µL of DMF was added. The reaction mixture was allowed to react at room temperature for 1 h. PSMA-1-VcMMAE was then purified by semi-preparative HPLC and lyophilized. Retention time: 17.6 min (**Figure S3A**). Mass spectrum (MS) (C_123_H_195_N_23_O_37_S), calculated: 2618.3; found: 2619 (M+1) (**Figure S3B**). Purified PSMA-1-VcMMAE (1.3 mg, 0.5 μmol) was dissolved in 0.5 mL PBS. The pH of the solution was adjusted to 7.5, then IRDye700 NHS ester (1.9 mg, 1 μmol, Li-Cor Inc.) in 0.5 mL PBS was added. The mixture was allowed to react at room temperature overnight. PSMA-1-MMAE-IR700 was then purified by HPLC. Yield: 1.6 mg, 76%. Retention time: 19.9 min (**Figure S4A**). Mass spectrum (MS) (ammonium salt: C_193_H_302_N_38_O_61_S_7_Si_3_), calculated: 4439; found: 971 ([M - 4 NH_4_ - C_14_H_30_NO_10_S_3_Si] / 4),1091 ([M - 4 NH_4_] / 4), 1295 ([M - 4 NH_4_ - C_14_H_30_NO_10_S_3_Si] / 3), 1456 ([M - 4 NH_4_] / 3), 1943 ([M - 4 NH_4_ - C_14_H_30_NO_10_S_3_Si] / 2) (**Figure S4B**).

### Cell culture

PSMA-positive PC3pip and PSMA-negative PC3flu cells were maintained in RPMI medium with 10% Fetal Bovine Serum at 37 °C and 5% CO_2_ under a humidified atmosphere. The cells were last sorted and checked by western blot in 2021.

### Competition binding studies

PC3pip cells (5 × 10^5^) was suspended in 200 uL of 50 mM Tris buffer, pH 7.5. The cells were incubated at 37 °C with different concentrations of PSMA-1-MMAE-IR700 or ZJ24 in the presence of 12 nM of ^3^H-ZJ24 for 1 h 57, 58, 60. The cells were then washed 3 times with cold PBS and cell-associated radioactivity was measured by scintillation counting. The concentration required to inhibit 50% of the binding (IC_50_) was determined by GraphPad Prism 3.0. Studies were performed in triplicate.

### Cathespin cleavage studies

Enzymatic cleavage study of PSMA-1-MMAE-IR700 was performed as previously described 60. PSMA-1-MMAE-IR700 was incubated with activated human liver cathespin (Anthens Research and Technology, Anthens, GA) at 37 °C. At different incubation time, 40 μL of the solution was placed into tubes loaded with 1 μL of 1 mM E64 protease inhibitor. The mixture was vortexed and then stored at -80 °C for future HPLC analysis. Studies were performed in triplicate.

### *In vitro* cellular uptake studies

Cells were plated on coverslips at about 60 - 70% confluency. Twenty-four hours later, cells were incubated with 50 nM of PSMA-1-MMAE-IR700. After incubation for various times (15 min, 30 min, 1 h and 4 h), cells were washed twice with cold RPMI 1640, fixed with 4% paraformaldehyde and counterstained with 4′,6-diamidino-2-phenylindole (DAPI) 57, 58, 60. Images were taken using a Leica DM4000B fluorescence microscope (Leica Biosystems, Buffalo Gove, IL) at 40×. Blocking experiments were performed by co-incubation of cells with 50 nM of PSMA-1-MMAE-IR700 and 100 × of PSMA-1 ligand. Studies were performed in triplicate.

### *In vitro* cytotoxicity studies

PC3pip and PC3flu cells were plated at 3,000 cells/well in 96-well plates. Twenty-four hours later, drugs (PSMA-1-MMAE-IR700 or PSMA-1-IR700) of different concentrations were added. After incubation at 37 °C in the dark for 24 h, cell viability was determined by CellTiter 96^®^ aqueous one solution cell proliferation assay using absorbance at 490 nm (Promega Biotech, Madison, MI). The concentration required to inhibit 50% of cell growth (IC_50_) was determined by GraphPad Prism 3.0. To test the cytotoxicity of PSMA-1-MMAE-IR700 with light irradiation, drugs at a final concentration of 5 nM were added to cells and incubated in the dark for 24 h. Cells were then washed 3 times with RMPI 1640 and then irradiated with 690 nm light (L690-66-60, Marubeni America Co, New York, NY). Cells were incubated in the dark for another 24 h. Cell viability was then determined by CellTiter 96^®^ aqueous one solution cell proliferation assay (Promega Biotech, Madison, MI). The coefficient drug interaction (CDI) 62, 63 was calculated as follows: CDI = AB / (A × B), where AB is the ratio of the absorbance of combination treatment groups (PSMA-1-MMAE-IR700 with light irradiation) to the absorbance of control groups; A or B is the absorbance of single treatment group (PSMA-1-MMAE-IR700 without light or PSMA-1-IR700 with light irradiation) to the absorbance of control groups. CDI < 1, = 1 and > 1 indicates synergistic, additive or antagonistic effect.

### *In vivo* fluorescence imaging studies

Animal experiments were approved by the animal care and use committee at Case Western Reserve University (IACUC#150033). Six to eight - week - old male Balb/c athymic nude mice (Jackson Laboratory, Bar Harbor, ME) were implanted subcutaneously with 1 × 10^6^ of PC3pip (right flank) and PC3flu (left flank) cells in 100 μL of matrigel. Animals were ready to use when tumor diameter reached 10 mm, about two weeks. Animals received 100 nmol/kg of PSMA-1-MMAE-IR700 through tail vein injection and were imaged at different time points by Maestro *In vivo* Image System (Perkin Elmer, Waltham, MI) using the yellow filter set (excitation 575-605 nm, emission 645 nm longpass). During imaging, mice were anesthetized by isoflurane. At 48 h post injection, mice were sacrificed and tissues such as heart, lung, liver, kidneys, stomach, tumors were harvested for *ex vivo* imaging. Multispectral images were unmixed into their component spectra and average fluorescence signals were quantified by creating regions of interest. Experiments were performed in 5 mice.

Orthotopic PC3pipGFP prostate cancer models were established as previously described using green fluorescence protein (GFP) transfected PC3pip cells 59. Mice bearing orthotopic PC3pipGFP tumors were injected with 100 nmol/kg of PSMA-1-MMAE-IR700. Mice were imaged at 1 h post injection by Maestro using the yellow filter set for PSMA-1-MMAE-IR700 and blue filter set for GFP (excitation 445-490 nm, emission 515 nm longpass). Mice were then euthanized and primary tumor was removed to expose lymph nodes. The mice were imaged again. Resected primary tumor and lymph nodes were snap-frozen in optimal cutting temperature (OCT) compound and sectioned. The slides were subjected to hematoxylin and eosin (H&E) staining, and adjacent set of slides were counter stained with DAPI and observed under Leica DM4000B fluorescence microscope at 10 × to visualize DAPI, GFP and PSMA-1-MMAE-IR700. Experiments were repeated in three mice.

### *In vivo* antitumor efficacy studies

The effect of PSMA-1-MMAE-IR700 were tested in mice bearing PC3pip tumors. Animals with tumor size at about 100 mm^3^ were used for the study (tumor volume = Length × width^2^ / 2). Animals were divided into 7 groups: (1) mice receiving PBS; (2) mice receiving 100 nmol/kg PSMA-1-MMAE-IR700 with PDT; (3) mice receiving equal doses of PSMA-1-MMAE-IR700 to group 2, but not receiving PDT; (4) Mice receiving equal doses of PSMA-1-IR700 to group 2 with PDT treatment; (5) Mice receiving equal doses of PSMA-1-IR700 to group 2 without PDT; (6) mice receiving equal doses of PSMA-1-IR700 to group 2 + free MMAE with PDT (MMAE normalized to that delivered by PSMA-1-MMAE-IR700); and (7) mice receiving equal doses of PSMA-1-IR700 to group 2 + free MMAE without PDT. Each group had 5 mice. Animals received drugs through tail vein injection on day 0, 4, 8, 12 and 16 and treated with 50 J/cm^2^ of 690 nm light at 1 h post-injection on these injection days. The dose and schedule was based on previous PSMA-1-VcMMAE work and were not optimized 60. Animals were imaged before and after PDT. Mice were monitored every other day for 90 days. Animals were euthanized when tumors became too large (diameter > 20 mm) or animals were moribund. Data were reported as body weight over time, tumor size over time and Kaplan-Meier survival plots.

### Immunofluorescent detection of apoptosis

Animals bearing PC3pip tumors were divided into 7 groups: (1) mice receiving PBS; (2) mice receiving 100 nmol/kg PSMA-1-MMAE-IR700 with PDT; (3) mice receiving equal doses of PSMA-1-MMAE-IR700 to group 2, but not receiving PDT; (4) Mice receiving equal doses of PSMA-1-IR700 to group 2 with PDT treatment; (5) Mice receiving equal doses of PSMA-1-IR700 to group 2 without PDT; (6) mice receiving equal dose of PSMA-1-IR700 to group 2 + free MMAE with PDT (MMAE normalized to that delivered by PSMA-1-MMAE-IR700); and (7) mice receiving equal dose of PSMA-1-IR700 to group 2 + free MMAE without PDT. Animals were treated with one single dose and were sacrificed at 4-day post treatment. Tumors were snap-frozen in OCT, cut into 10 μm thick sections and fixed on slides. Induction of apoptosis by the treatment was determined by rabbit polyclonal anti-Caspase-3 antibody (Abcam, Cambridge, UK). A goat anti-rabbit polyclonal antibody labeled by Alexa Fluor-594 was used as secondary antibody (Abcam, Cambridge, UK). The presence of apoptosis was determined by fluorescence images under Leica DM4000B fluorescence microscope at 10 ×. H&E staining of tumor tissues was performed in adjacent sections to check the histology of the tumors. Experiments were repeated in 5 mice.

### Statistics

Student *t*-test was used to compare inter-group differences. Kaplan-Meier survival data were analyzed by SAS 9.4 using log-rank tests. A p value < 0.05 was considered statistically significant for all comparisons.

## Results

### Chemistry and *in vitro* competition binding studies

To synthesize PSMA-1-MMAE-IR700 (**Figures 2A** and** S1**), we adopted our previous prodrug strategy and used a self-immolative cathespsin cleavable Vc linker to conjugate MMAE to PSMA-1-Cys-C6-Lys. IR700 was conjugated to the PSMA-1 ligand through reaction of the Lys amine (-NH2) with NHS ester as no IR700 release is required for light activation 58. PSMA-1-MMAE-IR700 had a maximum absorbance (λmax) at 690 nm (**Figure 2B**), which concurred with PSMA-1-IR700 58. In a competition binding assay (**Figure 2C**), PSMA-1-MMAE-IR700 showed IC_50_ at 2.44 ± 0.43 nM, which was 4.4 - fold greater than the related ligand ZJ24 (IC_50_ = 10.71 ± 0.89 nM) and similar to the PSMA-1 ligand 57, 58, 64. In the presence of cathepsin, PSMA-1-MMAE-IR700 degraded rapidly and release of MMAE was observed, supporting the prodrug strategy of PSMA-1-MMAE-IR700 (**Figures 2D** and** S5**). Stability studies found that PSMA-1-MMAE-IR700 was stable in PBS when incubated at 37 °C (**Figure S6A**). In mouse plasma, degradation of PSMA-1-MMAE-IR700 was observed with half-life at 17.2 ± 2.8 h (**Figure S6A**). Degradation of PSMA-1-MMAE-IR700 led to release of free MMAE (**Figure S6**).

### *In vitro* uptake studies

**Figure 3:**
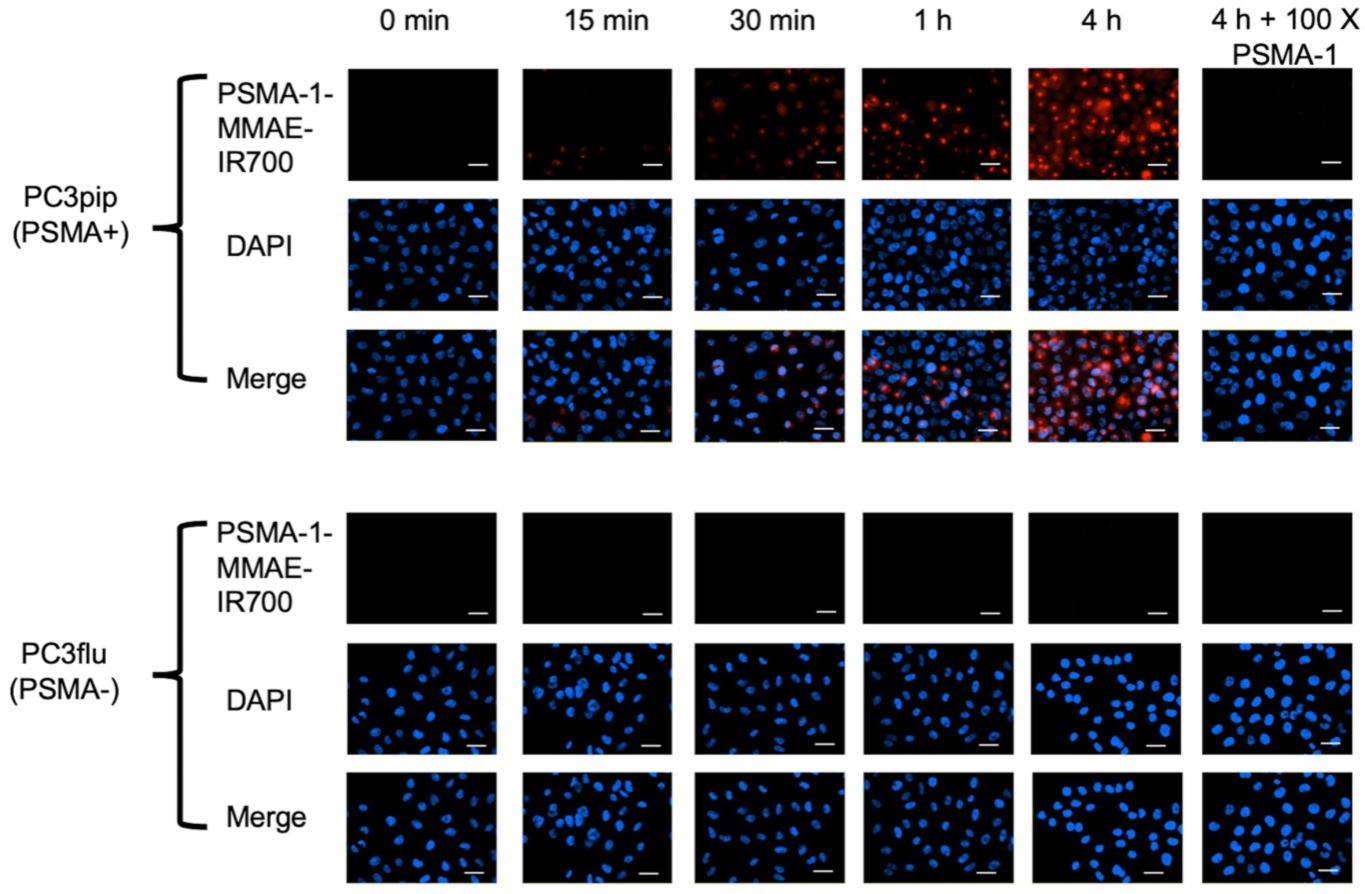
***In vitro* uptake studies of PSMA-1-MMAE-IR700 in PSMA-positive PC3pip and PSMA-negative PC3flu cells.** Cells were incubated with 50 nM of PSMA-1-MMAE-IR700 for various times. Blocking experiments were performed by co-incubation of cells with 50 nM of PSMA-1-MMAE-IR700 and 100 × of PSMA-1 ligand. Nuclei were stained by DAPI and false colored blue. PSMA-1-MMAE-IR700 signal was false colored red. Images were taken at 40 ×. Scale bar = 50 μm. Representative images are shown from three independent studies.

To determine the selectivity of PSMA-1-MMAE-IR700, uptake studies were performed using both PSMA-positive PC3pip and PSMA-negative PC3flu cells. Fluorescence signal in PC3pip cells was observed as early as 15 min after incubation with PSMA-1-MMAE-IR700 and the signal intensity increased with extended incubation time (**Figure 3**). In contrast, no fluorescence signal was observed in PC3flu cells. When PC3pip cells were co-incubated with PSMA-1-MMAE-IR700 and 100 × of PSMA-1 ligand, the fluorescence signal was completely blocked, indicating that the binding of PSMA-1-MMAE-IR700 was specific to PSMA. Further confocal images showed that the fluorescence from PSMA-1-MMAE-IR700 co-localized to the lysosome compartment of the cells where cathepsins are highly expressed 65 (**Figure S7**). The results concurred with our previous study with PSMA-1-VcMMAE-Cy5.5 60, suggesting the internalization of PSMA-1-MMAE-IR700 and its accumulation in lysosomes.

### *In vitro* cytotoxicity studies

**Figure 4:**
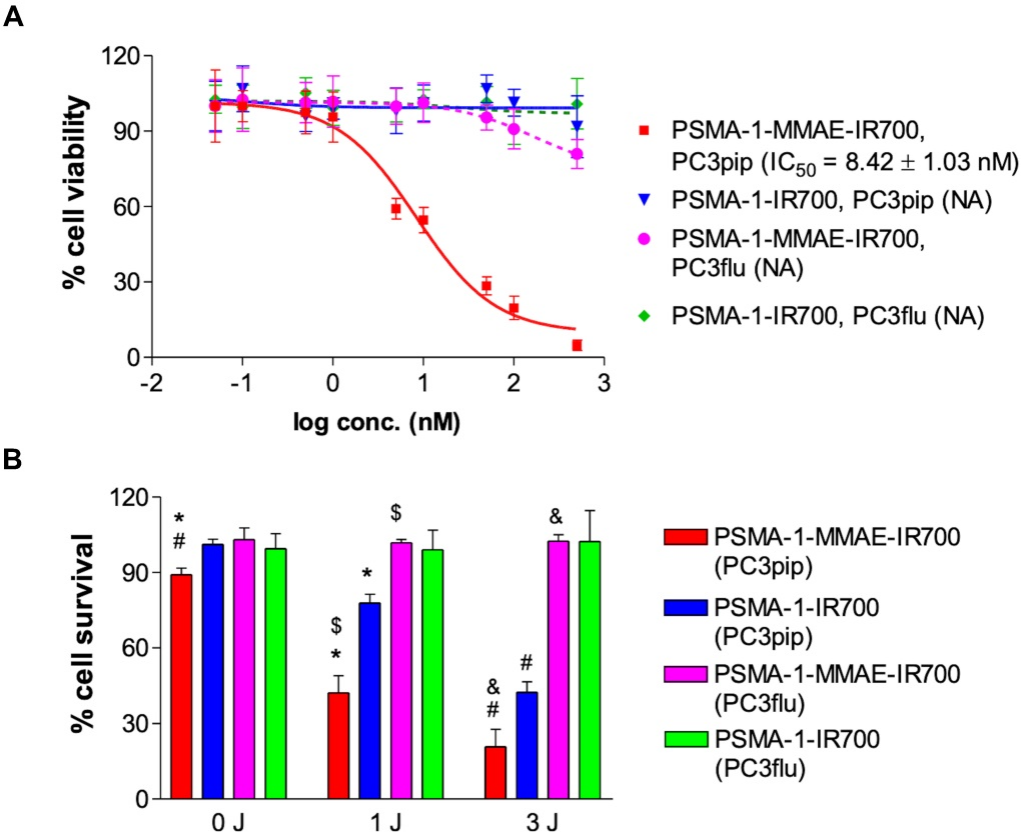
***In vitro* cytotoxicity of PSMA-1-MMAE-IR700. (A)** Dark cytotoxicity of PSMA-1-MMAE-IR700. Cells were incubated with drugs for 24 h in the dark, and then cell viability was determined. NA means IC_50_ value is not available. Values are mean ± SD of six replicates. **(B)** Cytotoxicity of PSMA-1-MMAE-IR700 with 690 nm light treatment. Cells were incubated with 5 nM of drugs for 24 h in the dark. Drugs were then washed off and cell were exposed to 1 J/cm^2^ or 3 J/cm^2^ light. Cell viability was measured 24 h later. Values are mean ± SD of six replicates. (*: P < 0.0001, PSMA-1-MMAE-IR700 with 1 J/cm^2^ light versus PSMA-1-MMAE-IR700 with no light, or PSMA-1-IR700 with 1 J/cm^2^ light. $: P < 0.0001, PSMA-1-MMAE-IR700 with 1 J/cm^2^ light to PC3pip cells versus that treatment to PC3flu cells. #: P < 0.0001, PSMA-1-MMAE-IR700 with 3 J/cm^2^ light versus PSMA-1-MMAE-IR700 with no light, or PSMA-1-IR700 with 3 J/cm^2^ light. &: P < 0.0001, PSMA-1-MMAE-IR700 with 3 J/cm^2^ light to PC3pip cells versus that treatment to PC3flu cells).

Cytotoxicity of PSMA-1-MMAE-IR700 was performed in both PSMA-positive PC3pip and PSMA-negative PC3flu cells to test if PSMA-1-MMAE-IR700 would selectively kill PSMA-positive cells. We first tested the cytotoxicity of PSMA-1-MMAE-IR700 and PSMA-1-IR700 in PC3pip and PC3flu cells without light treatment. After 24 h of incubation in the dark, PSMA-1-IR700 did not show any activity indicating that PSMA-1-IR700 is not toxic without light activation (**Figure 4A**). PSMA-1-MMAE-IR700 effectively killed PC3pip cells with an IC_50_ = 8.42 ± 1.03 nM, while it was much less potent in killing PC3flu cells and no IC_50_ value was obtained. Incubation of the cells with PSMA-1-MMAE-IR700 in the dark for 72 h showed that it was 50-fold more effective for killing PC3pip cells than for PC3flu cells (**Figure S8**). Our results suggest that PSMA-1-MMAE-IR700 selectively delivers MMAE to PC3pip cells leading to effective cell death. To test if combination of MMAE-based chemotherapy and PDT using PSMA-1-MMAE-IR700 would enhance the cytotoxicity, PC3pip and PC3flu cells were incubated with 5 nM of different targeted agents for 24 h, and then cells were washed and treated with or without light. **Figure S9** shows that exposure of PC3pip and PC3flu cells to 690 nm light without incubation with drugs does not kill PSMA+ or PSMA- cancer cells. PSMA-1-MMAE-IR700 with light treatment (1 J/cm^2^ and 3 J/cm^2^) significantly enhanced the cytotoxicity to PC3pip cells as compared to PSMA-1-IR700 with light treatment and compared to PSMA-1-MMAE-IR700 without light treatment (0 J/cm^2^) (**Figure 4B**). In addition, the cytotoxicity of PSMA-1-MMAE-IR700 increased when the dose of light was increased from 1 J/cm^2^ to 3 J/cm^2^. CDI was 0.423 / (0.778 × 0.892) = 0.609 and 0.208 / (0.424 × 0.892) = 0.550 at 1 J/cm^2^ and 3 J/cm^2^ light irradiation, respectively, suggesting that combination of PSMA-targeted PDT and MMAE caused synergistic effects. In contrast to the cytotoxicity observed in PC3pip cells, no effective cell killing was observed in PC3flu cells when the cells were treated with 5 nM of PSMA-1-MMAE-IR700 or PSMA-1-IR700 with or without light, indicating the cell killing effect is selective to PSMA expression.

### *In vivo* fluorescence imaging results

**Figure 5:**
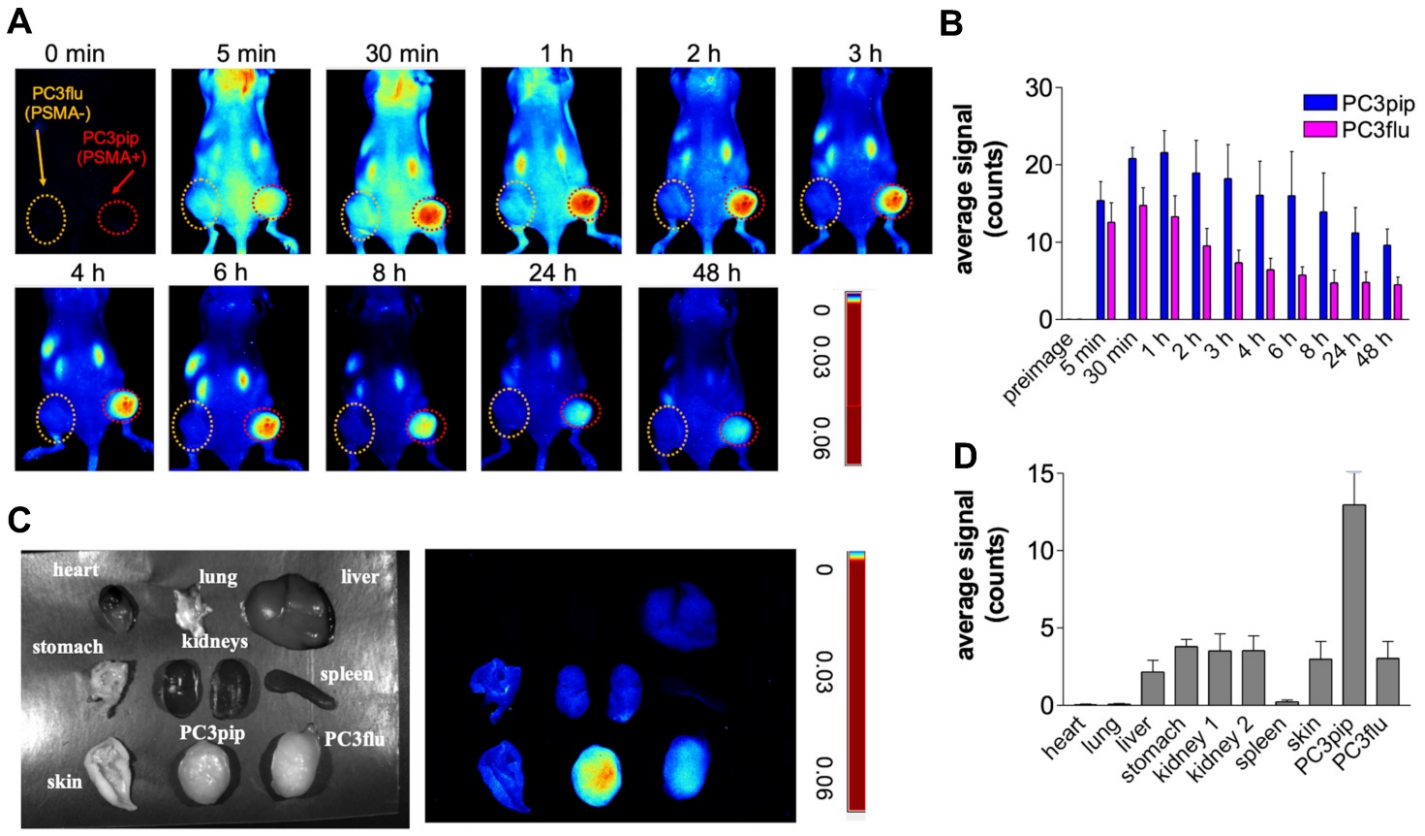
***In vivo* fluorescence images of PSMA-1-MMAE-IR700. (A)** *In vivo* Maestro imaging of a typical mouse bearing heterotopic PC3pip and PC3flu tumors treated with 100 nmol/kg of PSMA-1-MMAE-IR700 delivered through *i.v.* injection. Representative images are shown of n = 5. Selective uptake was observed in PC3pip tumors. **(B)** Quantification of fluorescent signal intensity in PC3pip and PC3flu tumors. Values are mean ± SD of 5 animals. **(C)** *Ex vivo* imaging of mouse organs at 48 h post injection. Fluorescence from PC3pip tumors was significantly higher than in other organs. Representative images are shown of n = 5. **(D)** Quantification of fluorescent signal intensity in tissues. Values are mean ± SD of 5 animals.

To evaluate the selectivity of PSMA-1-MMAE-IR700, *in vivo* fluorescence imaging was performed in mice bearing both PC3pip and PC3flu tumors. PSMA-1-MMAE-IR700 (100 nmol/kg) was administered through tail vein injection, and mice were imaged at various time points. As shown in **Figures 5A-B**, selective uptake was observed in PC3pip tumors. The time to reach peak uptake in PC3pip tumors was 1 h post injection. At 4 h post injection, the fluorescence signal in PC3 pip tumor was 3.8 - fold higher (19.6 ± 4.4 counts) than that in PC3flu tumor (5.2 ± 1.5 counts); at 48 h post injection, the difference between the fluorescence signal from the two tumors was 2.9 - fold (9.6 ± 2.1 counts on PC3pip tumors *vs* 3.3 ± 1.3 counts on PC3flu tumors) **(Figure 5B)**. *Ex vivo* imaging at 48 h post injection showed that fluorescence was mainly retained in the PSMA-positive PC3pip tumor (**Figures 5C-D).** Minimal fluorescence was observed in PSMA-negative PC3flu tumor, liver, spleen, lung, kidneys, heart, lung, skin, and stomach.

**Figure 6:**
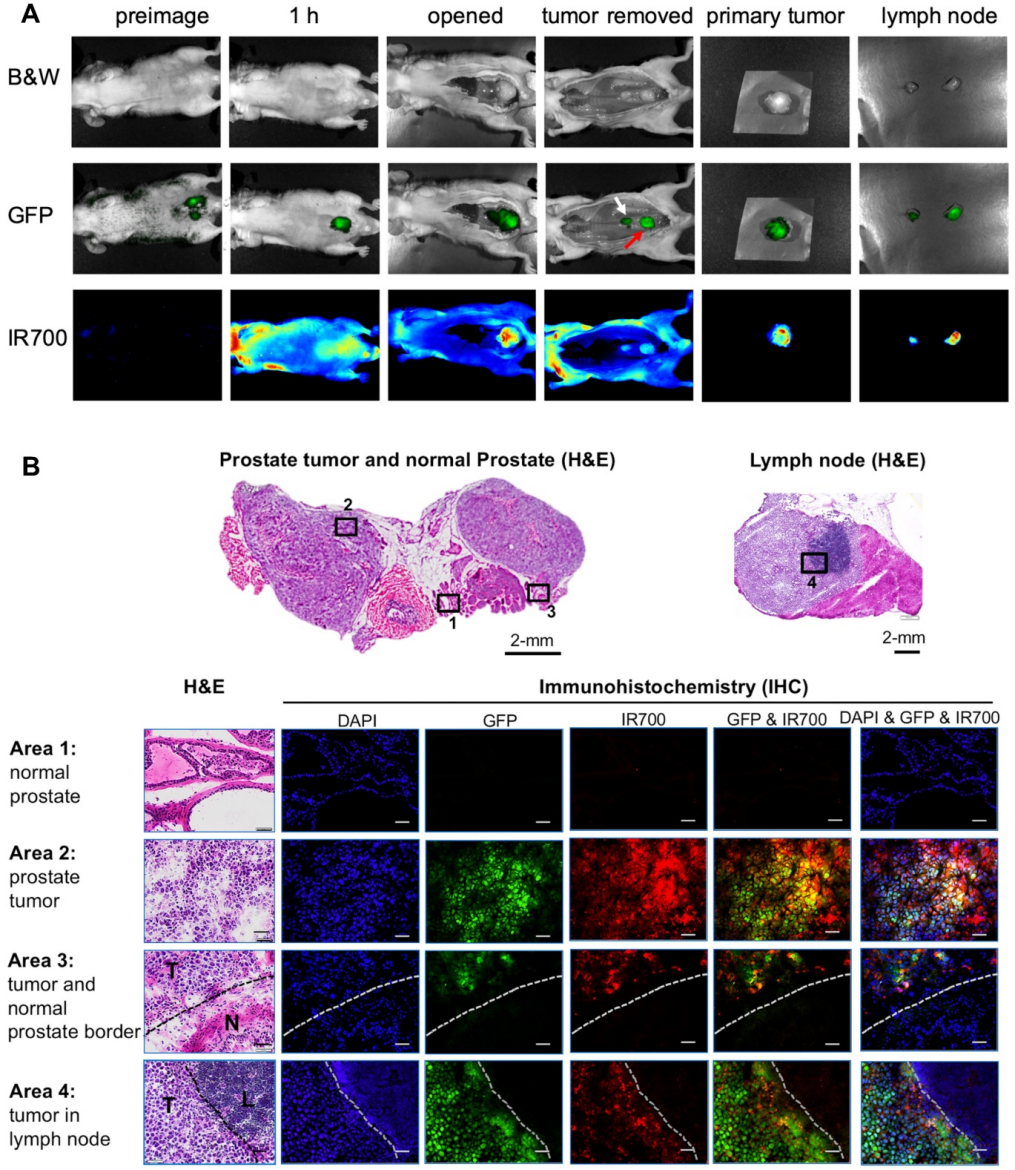
** Detection of primary orthotopic prostate tumor and lymph node metastases by PSMA-1-MMAE-IR700.** Representative images are shown from 3 animals. **(A)** *In vivo* and *ex vivo* fluorescence image of PSMA-1-MMAE-IR700 in mice bearing orthotopic PC3pipGFP tumor. Mice received 100 nmol of PSMA-1-MMAE-IR700 through tail vein injection. Images were taken at 1 h post injection. White arrow indicates lymph node, and red arrow indicates residual primary tumor. **(B)** Histological analysis of dissected primary tumor and lymph nodes. Presence of tumor cells was confirmed by H&E staining, GFP signal (false colored green), and IR700 signal from PSMA-1-MMAE-IR700 (false colored red). Nuclear stain, DAPI, is false colored blue. Scale bar = 100 μm in the lower panel. Black or white dashed lines indicate the borderline between normal tissue and cancer tissues. “T” is for tumor tissues; “N” is for normal prostate; and “L” is for lymphocytes.

As we reported previously, the orthotopic PC3pip prostate cancer mouse model can develop tumor metastases to lymph nodes 59. To test if PSMA-1-MMAE-IR700 can detect lymph node metastases, we performed fluorescence imaging in mice bearing PC3pipGFP tumors. Twenty-one days following orthotopic implantation of PC3pip cells into the prostate gland, mice received 100 nmol/kg PSMA-1-MMAE-IR700. At 1 hour post injection (peak tumor accumulation time determined above), bright fluorescence signal from PSMA-1-MMAE-IR700 was observed in primary orthotopic PC3pipGFP tumors and the signal correlated with the GFP fluorescence signal in the tumor (**Figure 6A**). Removal of the primary tumor allowed visualization of enlarged lymph nodes. GFP signal in the lymph nodes proved presence of tumor in the lymph nodes. Fluorescence signal from PSMA-1-MMAE-IR700 corresponded to the GFP in the lymph nodes. *Ex vivo* imaging of tissues at 1 h post injection showed that PSMA-1-MMAE-IR700 fluorescence was only observed in the tumor and kidneys (**Figure S10**). The signal in kidneys dropped much more rapidly from 1 h to 48 h (**Figures 5C-D**) as compared to the signals in PC3pip tumors, suggesting that the signal in kidneys was mainly due to renal clearance of PSMA-1-MMAE-IR700. Further histological analysis was performed in the primary tumor and lymph node (**Figure 6B**). Fluorescence signal from PSMA-1-MMAE-IR700 was only observed in cancer tissues, but not in normal prostate or normal lymphocytes. PSMA-1-MMAE-IR700 fluorescence signal was highly co-localized to GFP signal from the tumor. In addition, PSMA-1-MMAE-IR700 was able to define the borders between the cancer tissue and normal tissue.

### *In vivo* antitumor activity studies

**Figure 7:**
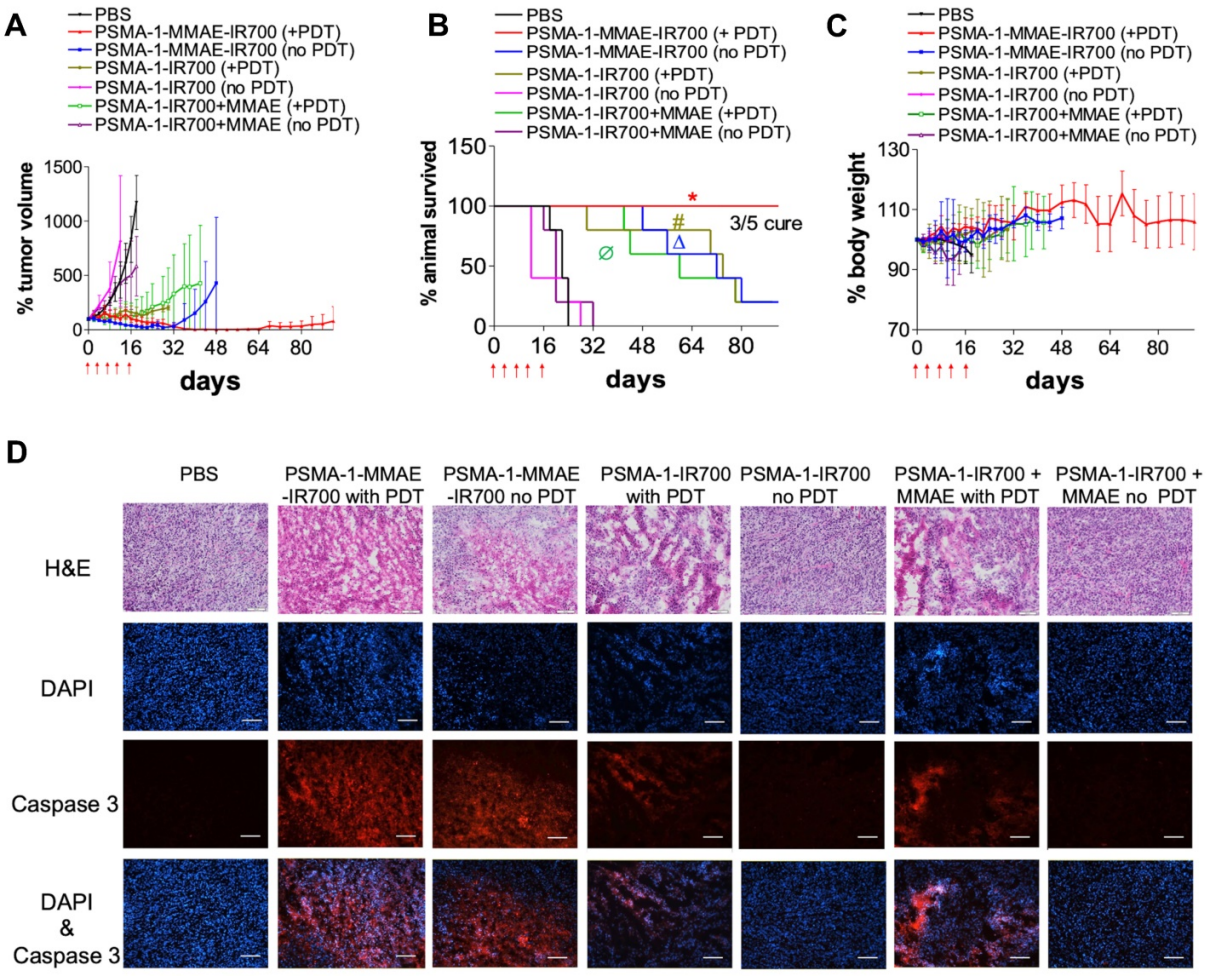
***In vivo* antitumor activity of PSMA-1-MMAE-IR700 in mice bearing heterotopic PC3pip tumors.** For survival experiments, mice received drugs through tail vein injection. PDT was performed at 1 hour post injection. Treatment was scheduled every 4 days with a total of five doses as indicated by the red arrows. Each group had 5 mice. For tumor growth curves and body weight curves, values are mean ± SD of 5 animals. The plots stopped when animals died during the experiments since values are represent as mean ± SD of 5 animals. **(A)** Tumor growth curves of mice. **(B)** Kaplan-Meier survival curves of treated mice. (*, P < 0.05, PSMA-1-MMAE-IR700+PDT versus the other 6 groups. Δ, P < 0.05, PSMA-1-MMAE-IR700 without PDT versus PBS, PSMA-1-IR700 without PDT and PSMA-1-IR700 + MMAE without PDT. #, P < 0.05, PSMA-1-IR700 with PDT versus PBS, PSMA-1-IR700 without PDT and PSMA-1-IR700 + MMAE without PDT; Ø, P < 0.05, PSMA-1-IR700 + MMAE with PDT versus PBS, PSMA-1-IR700 without PDT and PSMA-1-IR700 + MMAE without PDT). P values between different groups are summarized in **Table S1**. **(C)** Body weight changes of mice treated with PSMA-1-VcMMAE. **(D)** Induction of apoptosis by the treatment. Tumors were dissected at 4 days post treatment and examined by H&E staining and caspase-3 assay. DAPI is false colored blue and caspase-3 is false colored red. Scale bar = 100 μm. Pictures are representative images of five mice.

The effectiveness of PSMA-1-MMAE-IR700 to eliminate prostate tumors was performed in mice bearing PC3pip tumors. Mice received 100 nmol of PSMA-1-MMAE-IR700 through tail vein injection every 4 days with a total of 5 doses. Mice were irradiated by 50 J/cm^2^ of 690 nm light at the peak tumor accumulation of PSMA-1-MMAE-IR700, which was 1 h post injection. Controls included *i.v.* administration of PBS with no light treatment, 100 nmol/kg of PSMA-1-MMAE-IR700 with 50 J/cm^2^ of 690 nm light, 100 nmol/kg of PSMA-1-MMAE-IR700 with no light treatment, 100 nmol/kg of PSMA-1-IR700 with 50 J/cm^2^ of 690 nm light, 100 nmol of PSMA-1-IR700 with no light treatment, co-injection of 100 nmol/kg of PSMA-1-IR700 and 100 nmol/kg of free MMAE with 50 J/cm^2^ of 690 nm light, and co-injection of 100 nmol/kg of PSMA-1-IR700 and 100 nmol/kg of free MMAE with no light treatment. As shown in **Figures 7A-B** and **Figure S11**, PSMA-1-IR700 with no light and PSMA-1-IR700 + MMAE with no light failed to inhibit tumor growth and extend animal survival. In contrast to the group that received PSMA-1-IR700 + MMAE without irradiation, PSMA-1-IR700 with light irradiation and PSMA-1-IR700 + MMAE with light irradiation showed the ability to inhibit tumor growth. PDT caused tumor swelling in some mice (**Figure S11**). Most of the mice in these two PDT groups initially displayed tumor growth inhibition, but after the treatment period the tumor started to grow again leading to animal death, with one mouse in the PSMA-1-IR700 + PDT group dying on day 32 of the treatment** (Figures 7A-B** and **Figure S11)**. These data indicate that PDT alone is not enough to kill the tumors completely. PSMA-1-MMAE-IR700 without light significantly inhibited tumor growth and prolonged animal survival, indicating that targeted delivery of MMAE improved antitumor activity. The greatest tumor growth inhibition was found in the animals receiving PSMA-1-MMAE-IR700 with light irradiation. During PSMA-1-MMAE-IR700 + PDT treatment, initial tumor swelling was observed in some mice, resulting in slightly larger initial tumor sizes than those measured after treatment with PSMA-1-MMAE-IR700 without PDT. Importantly, the sizes of PSMA-1-MMAE-IR700 + PDT treated tumors continued to shrink even when the treatment ceased. In contrast, the tumors of the mice receiving PSMA-1-MMAE-IR700 without PDT treatment started to grow when the treatment stopped. Between day 40 to day 60, all 5 mice that received PSMA-1-MMAE-IR700 with PDT were tumor free. After day 60, tumor started to grow back on 2 of the mice, and the other 3 mice remained tumor free during the 90-day experimental period. Although we were not able to obtain the CDI values as the control mice started to die as early as on day 18, our data demonstrated that PSMA-1-MMAE-IR700 + PDT significantly inhibited tumor growth (**Figure 7A**) and extended animal survival (**Figure 7B**) as compared to the other 6 groups (**Table S1**). Therefore, PSMA-1-MMAE-IR700 with light improved the treatment outcome of PSMA-1-IR700 with light irradiation and PSMA-1-MMAE-IR700 with no light irradiation. In addition, the treatment did not cause any body weight loss (**Figure 7C**). Further histological analysis of major organs in mice treated with PSMA-1-MMAE-IR700 on day 30 did not show any considerable microscopic changes in major organs, such as liver, lung, heart, spleen, kidney, salivary gland and prostate, indicating the treatment did not cause systemic toxicity (**Figure S12**).

### Induction of apoptosis by treatment

To evaluate the apoptosis caused by the treatment, mice bearing PC3pip tumors were treated with drugs and tumors were collected four days after the treatment, sectioned, and examined by H&E and caspase 3 assay (**Figure 7D**). The histology of the tumors treated with PSMA-1-IR700 without PDT and PSMA-1-IR700+MMAE without PDT remained the same as the PBS-treated control tumors. No immunofluorescence signal related to apoptosis was observed in these tumors. Treatment of the tumors with PSMA-1-IR700 with PDT or PSMA-1-IR700+MMAE with PDT caused significant necrotic damage (H&E) to the tumor tissue. The Caspase 3 assay revealed large portion of apoptotic lesions in the tumors. Similar observations were found in the tumors treated with PSMA-1-MMAE-IR700 without PDT. The most aggressive necrotic damage and apoptosis was observed in tumors receiving PSMA-1-MMAE-IR700 with PDT, suggesting that combination of targeted PDT with targeted chemotherapy enhanced the treatment effect.

## Discussion

Prostate cancer is highly heterogeneous 66, 67, which will affect treatment response, drug resistance and clinical outcome. The use of combination therapies with different mechanisms of action will offer potential advantages over a single therapy and it can be an effective way to deal with the 'heterogeneity' of cancer cells 68, 69. However, this is not a simple approach because different drugs may have different pharmacokinetics and do not necessarily get to the tumor at the same time and the drug can also go to other tissues in the body causing side effects. The use of anticancer drugs is therefore limited by unwanted side effects. To overcome these problems, we have developed a multifunctional molecule named PSMA-1-MMAE-IR700 that combines chemotherapy, PDT, and imaging in a single molecule that is targeted to PSMA. PSMA is over expressed almost exclusively on prostate cancer (**Figure 2A**). MMAE was selected as the chemotherapeutic drug because of its high potency 70, its wide use in antibody drug conjugates 71, its synergy with PDT 72 and the fact that we have successfully targeted it to PSMA for the treatment of prostate cancer with a greater therapeutic index when compared to a PSMA-targeted antibody drug conjugate using MMAE 60. IR700 was selected as the photosensitizer due to its good water-solubility, its high fluorescence quantum yield and singlet oxygen yield 64, its long wavelength absorption (690 nm), and the fact that a cetuximab-IR700 conjugate (RM-1929) has been approved in Japan for local regional treatment of head and neck cancer 73-75. Conjugation of MMAE and IR700 to the PSMA-1 ligand did not reduce the binding affinity of PSMA-1-MMAE-IR700 to PSMA (**Figure 2C**). Stability studies showed that while PSMA-1-MMAE-IR700 was stable in PBS, it degraded in mouse plasma with a half-life at 17.2 ± 2.8 h (**Figure S6**). This result is in accordance with Cazzamalli *et al.*'s report 76. The fluorescence from IR700 allowed visualization of uptake studies of PSMA-1-MMAE-IR700. PSMA-1-MMAE-IR700 showed selective uptake in PSMA-positive PC3pip cells, but not in PSMA-negative PC3flu cells, and its binding was specific to PSMA receptor on the cells as indicated by the blocking experiment with excess PSMA-1 ligand (**Figure 3**). Once it entered the cells, PSMA-1-MMAE-IR700 was almost exclusively located in the lysosomal compartment (**Figure S7**). These results are consistent with our previous studies 57, 58, 60. In *in vitro* cytotoxicity studies, PSMA-1-MMAE-IR700 without light activation selectively killed PC3pip cells, likely due to protease release of MMAE, which does not require PDT (**Figure 4**). Furthermore, PSMA-1-MMAE-IR700 with light treatment showed PDT efficacy and greater cytotoxicity than PSMA-1-MMAE-IR700 without light and PSMA-1-IR700 with light. These data suggested that the combination of PDT and MMAE in a single molecule was the most effective at killing PSMA-positive cancer cells. The combination appeared to be synergistic and CDIs of < 0.6 were observed. *In vivo* biodistribution studies showed that PSMA-1-MMAE-IR700 preferentially accumulated in PC3pip tumors (**Figure 5**). At 4 h post injection, the fluorescent signal in PC3pip tumors was 3.8 - fold higher than in PC3flu tumors. *Ex vivo* images at 48 h post injection showed that the fluorescent signal in PC3pip tumor was more than 3 - fold higher than in kidneys, liver and other organs, which indicates low off-target accumulation of PSMA-1-MMAE-IR700. Pharmacokinetic studies based on the fluorescence of PSMA-1-MMAE-IR700 showed that PSMA-1-MMAE-IR700 cleared quickly from the blood, after 4 h post injection almost all drug was cleared (**Figure S13**). Although PSMA-1-MMAE-IR700 degraded in mouse plasma, its degradation is much slower than its clearance from the blood stream, therefore, there shouldn't be concerns about toxicity from released MMAE. In fact, no body weight loss nor damage to major organs were observed after the treatment (**Figures 7C** and** S12**). In *in vivo* antitumor activity studies, PSMA-1-MMAE-IR700 demonstrated significantly greater efficacy for PC3pip tumors than single drug treatments (PSMA-1-MMAE-IR700 without PDT or PSMA-1-IR700 with PDT) (**Figure 7**). More noteworthy, PSMA-1-MMAE-IR700 showed improved antitumor activity compared to co-administration of individual drugs (PSMA-1-IR700+MMAE with PDT), addressing the importance of simultaneous drug delivery to cancers. PSMA-1-MMAE-IR700 at 100 nmol/kg with light irradiation resulted in a 60% cure rate for tumors. In previous studies, to reach 60% cure, a dose of 3820 nmol/kg of PSMA-1-VcMMAE was used, therefore, combination of PDT and chemotherapy significantly reduced the dose of chemotherapy required. A caspase 3 assay showed that tumors treated with PSMA-1-MMAE-IR700 + PDT had more apoptosis than tumors in the other treatment groups (**Figure 7D**), indicating that PSMA-1-MMAE-IR700 + PDT is the most efficacious treatment. It is speculated that PSMA-1-MMAE-IR700 + PDT may kill significantly more cancer cells than other treatment groups, leading to slower regrowth, *i.e.* less residual tumor cells, and/or therapeutic cures.

In the past few years, approaches that combine PDT and chemotherapy have been reported. Most of them use nanoparticles to delivery PDT and chemotherapy 77-81. For example, He *et al.* reported core-shell nanoscale coordination polymer (NCP) that carries oxaliplatin and pyrolipid 77. Synergistic effects were observed in the treatment of colorectal cancer. This NCP can also elicit antitumor immunity. Wang *et al.* loaded Pt(IV) anticancer drug and Chlorin e6 into layered double hydroxide nanoparticles and found that the nanobybrid showed synergistic cell killing and could overcome cisplatin-resistance 82. Attempts have also been made to add targeting moieties such as folic acid to the nanoparticles to actively delivery drugs to cancer cells 83-85. Although approaches have been made in nanoparticle-based drug delivery, the diversity and complexity of nanoparticles complicate the drug regulation pathway. Nanoparticles are known to cause immunogenic response, have unwanted toxicity and have inherent batch-to-batch variation 86-88. Compared to nanoparticles, small molecules can be synthesized more cost-effectively and have a lower probability of causing an immunogenic response. Combretastaine A-4 89 and paclitaxel 90 have been conjugated to phthalocyanine (Pc) to achieve PDT and chemotherapy in one molecule. More recently, folic acid has been conjugated to the molecules to target folate receptor 89, 91. Ito *et al.* successfully conjugated both IR700 and maytansinoid (DM1) to trastuzumab 92. These few examples showed that combination of targeting, PDT and chemotherapy can be achieved in one simple molecule or in the antibody-drug conjugate and are beginning to define a new class of combination therapy agents for cancers.

PSMA is an attractive target for the treatment of prostate. A few PSMA-targeting multifunctional molecules have been reported. Kumar *et al.* combined ^68^Ga-PET imaging and DM1 to a PSMA targeting ligand for prostate cancer imaging and therapy 93. Lutjel *et al.* conjugated IR700 and ^111^In to an anti-PSMA monoclonal antibody D2B for radionuclide and fluorescence imaging and PDT of prostate cancer 94. More recently, Derks *et al.* reported a PSMA ligand-based multimodal ^111^In-IRDy700dx-PSMA ligand conjugate 95. These approaches mainly focused on the combination of imaging with one treatment option. Our molecule is the first example that combines both chemotherapy and PDT in a single targeted small molecule for a dual-therapeutic approach to combat prostate cancer. The selective targeting and rapid clearance of the molecule should dramatically reduce off-target toxicity while simultaneously increasing anti-cancer efficacy. For localized prostate cancer, minimally invasive fiber optics have been developed to irradiate the prostate gland with light, e.g. TOOKAD 96, 97, providing the needed infrastructure for implementation of the PDT approach. In addition to localized NIR light irradiation, the efficacy of PSMA-1-MMAE-IR700 can be extended to systemic cell killing by local release of active MMAE, which will overcome the problem that PDT cannot be used to treat large tumors due to limited light penetration 98. On the other hand, PDT will reduce the needed dose of the chemotherapeutic drug 57, 58, therefore further reducing dose-related toxicity of MMAE. Compared to current clinical protocols for combination therapy of PDT and chemotherapy, our approach selectively delivers both drugs to cancer cells, reducing off-target toxicity related to untargeted drugs and achieving enhanced synergistic antitumor activity. Furthermore, the PDT agent, IR700, emits light at 700 nm when irradiated by 690 nm light. We have demonstrated that PSMA-1-MMAE-IR700 can identify cancer tissues, including metastases to lymph nodes, and delineate tumor margins (**Figure 6**), which further expands its use for fluorescence imaging and image-guided surgery for prostate cancer. It has been reported that IR700 can be imaged by a clinically available imaging instrument, LIGHTVISION (Shimaduzu, Japan) 99. Therefore, PSMA-1-MMAE-IR700 can help surgeons visualize the tumor and resect tumors during surgery. We have demonstrated that PDT is an effective adjuvant therapy after fluorescence image guided surgery (FIGS) 59. In the case of PSMA-1-MMAE-IR700, it can offer both adjuvant PDT and chemotherapy after FIGS to eradicate any unresected cancer cells, resulting in complete tumor removal. In previous studies, PSMA-1-VcMMAE has shown the ability to prevent tumor metastases 60. In this study, PSMA-1-MMAE-IR700 showed the ability to accumulate in tumor metastases in lymph nodes, which indicates that the drug may be effective in combating tumor metastases. In addition, both MMAE 100 and PDT 25-27, 101 have been reported to initiate a post-treatment immune response against tumor cells. The immune stimulation by our approach may further prevent the metastasis of the disease. Future works are needed to validate the use of PSMA-1-MMAE-IR700 for FIGS and the immune response caused by PSMA-1-MMAE-IR700 treatment using an immunocompetent mouse model of prostate cancer that overexpresses PSMA.

## Conclusions

In conclusion, we have synthesized a multifuncational theranostic molecule for simultaneous and targeted delivery of both PDT and chemotherapy to prostate cancer cells. The multifunctional molecule showed selective uptake in PSMA-positive tumors and significantly enhanced (synergistic) antitumor activity was observed as compared to individual treatment with PDT or chemotherapy alone. It can be used in the operating room to help surgeons detect tumors using real-time FIGS, and provide PDT and chemotherapy to kill any unresected cancer cells. It is also possible that the molecule can be used directly on prostate cancer patients that are not suitable for surgery, providing PDT and chemotherapy to cancer tissues. If successful, the new combined approach will provide a new treatment option for patients with high-risk localized prostate cancer. Further studies are required to better understand the impact of the dual treatment to stimulate the immune system and prevent metastatic cancer progression.

## Supplementary Material

Supplementary figures and table.

## Abbreviations

PDT: photodynamic therapy
PSMA: prostate specific membrane antigen
MMAE: monomethyl auristatin E
PSA: prostate specific antigen
ADT: androgen deprivation therapy
ROS: reactive oxygen species
FDA: Food and Drug Administration
PET: positron emission tomography
HPLC: high performance liquid chromatography
MS: mass spectrum
DAPI: 4′,6-diamidino-2-phenylindole
CDI: coefficient drug interaction
H&E staining: hematoxylin and eosin staining
GFP: green fluorescence protein
