# Supplementary material for: A low molecular weight multifunctional theranostic molecule for the treatment of prostate cancer
Source: Theranostics. 2022 Feb 21;12(5):2335–50. doi: 10.7150/thno.68715 (PMC8899574; doi:10.7150/thno.68715)
Supplement: Supplementary file 1 — Supplementary figures and table. [file thnov12p2335s1.pdf]

A low molecular weight multifunctional theranostic molecule for the treatment of prostate cancer

Xinning Wang[1,*], Rongcan Sun[2], Jing Wang[2], Jing Li[3], Ethan Walker[1], Aditi Shirke[1], Gopolakrishnan Ramamurthy[2], Lingpeng Shan[4], Dong Luo[2], Lauren Carmon[5], and James P. Basilion[1,2,*]

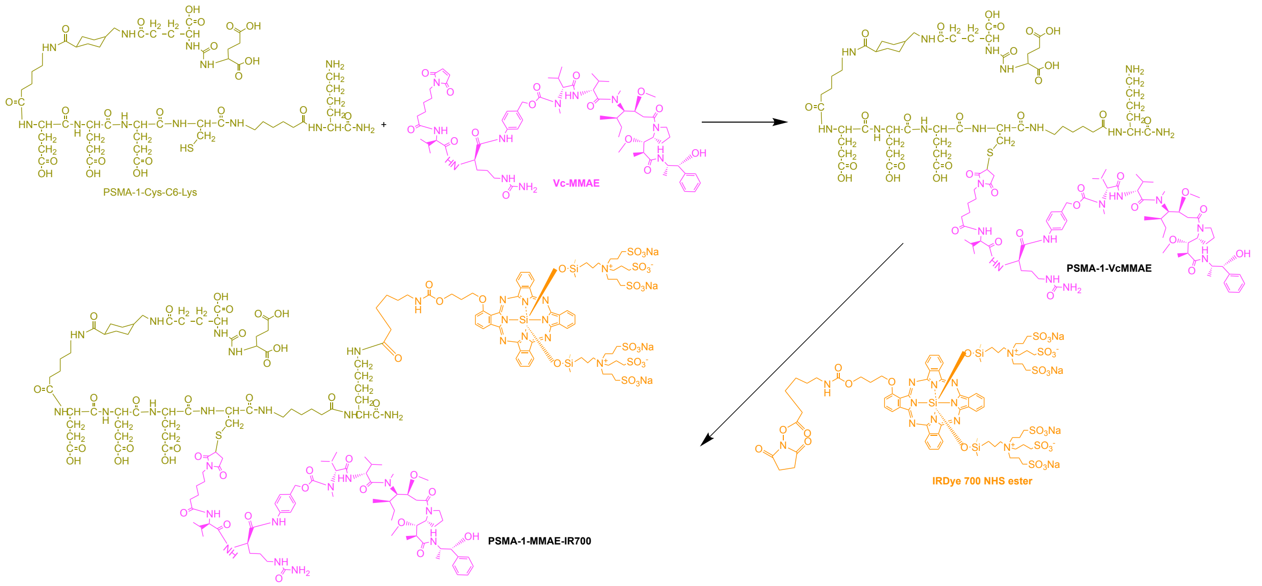

Figure S1. Synthetic scheme of PSMA-1-MMAE-IR700.

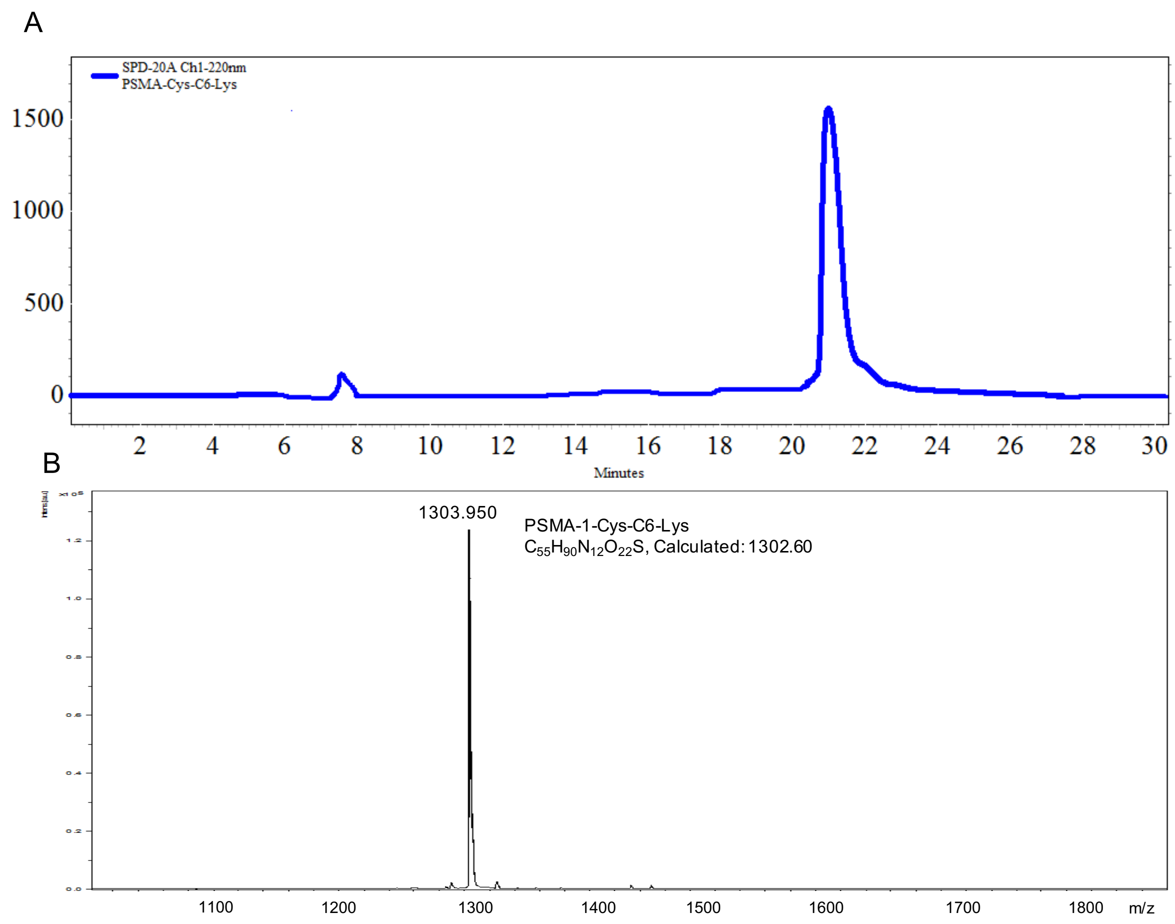

Figure S2. Characterization of PSMA-1-Cys-C6-Lys. (A) HPLC of purified PSMA-1-Cys-C6-Lys. HPLC gradient: 10-40% acetonitrile against 0.1% trifluoroacetic acid in 30 min. Retention time at 21.3 min. (B) Mass spectrum of PSMA-1-Cys-C6-Lys. MS spectrum was performed in positive mode. A peak at 1303.950 (M+1) was found.

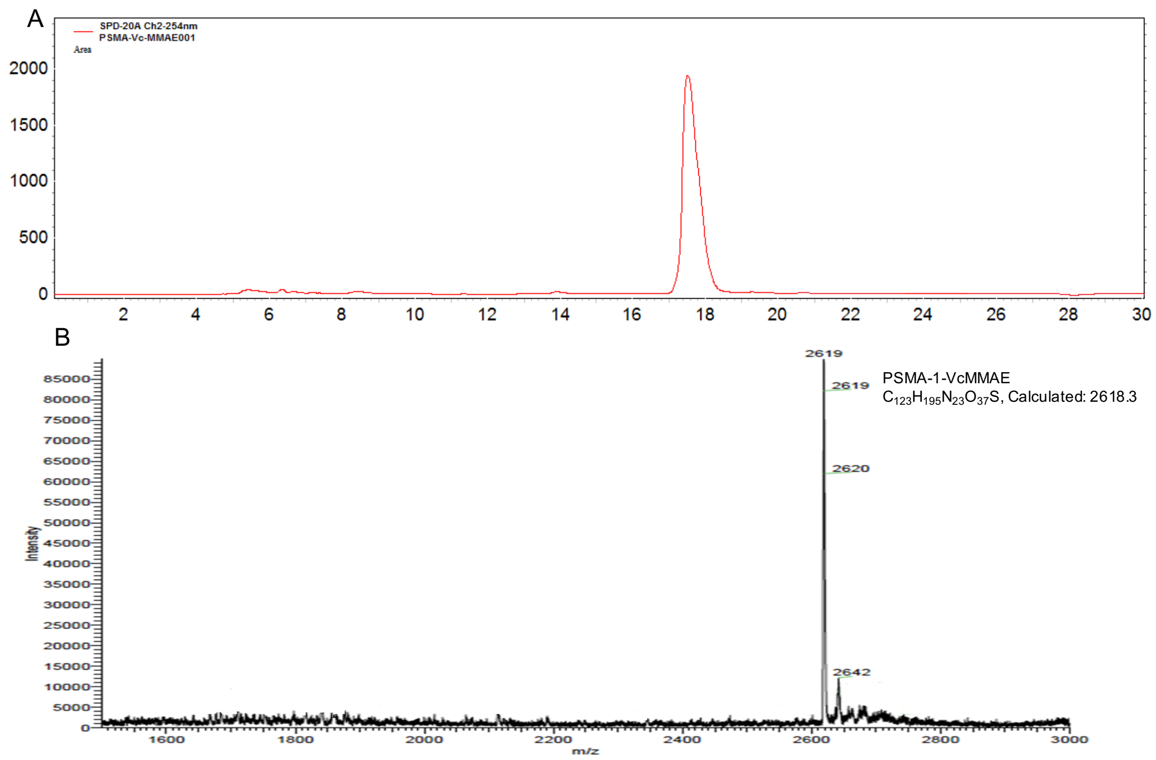

Figure S3. Characterization of PSMA-1-VcMMAE. (A) HPLC of purified PSMA-1-VcMMAE. HPLC gradient: 10-90% acetonitrile against 0.1% trifluoroacetic acid in 30 min. Retention time at 17.6 min. (B) Mass spectrum of PSMA-1-VcMMAE. MS spectrum was performed in positive mode. A peak at 2619 (M+1) was found.

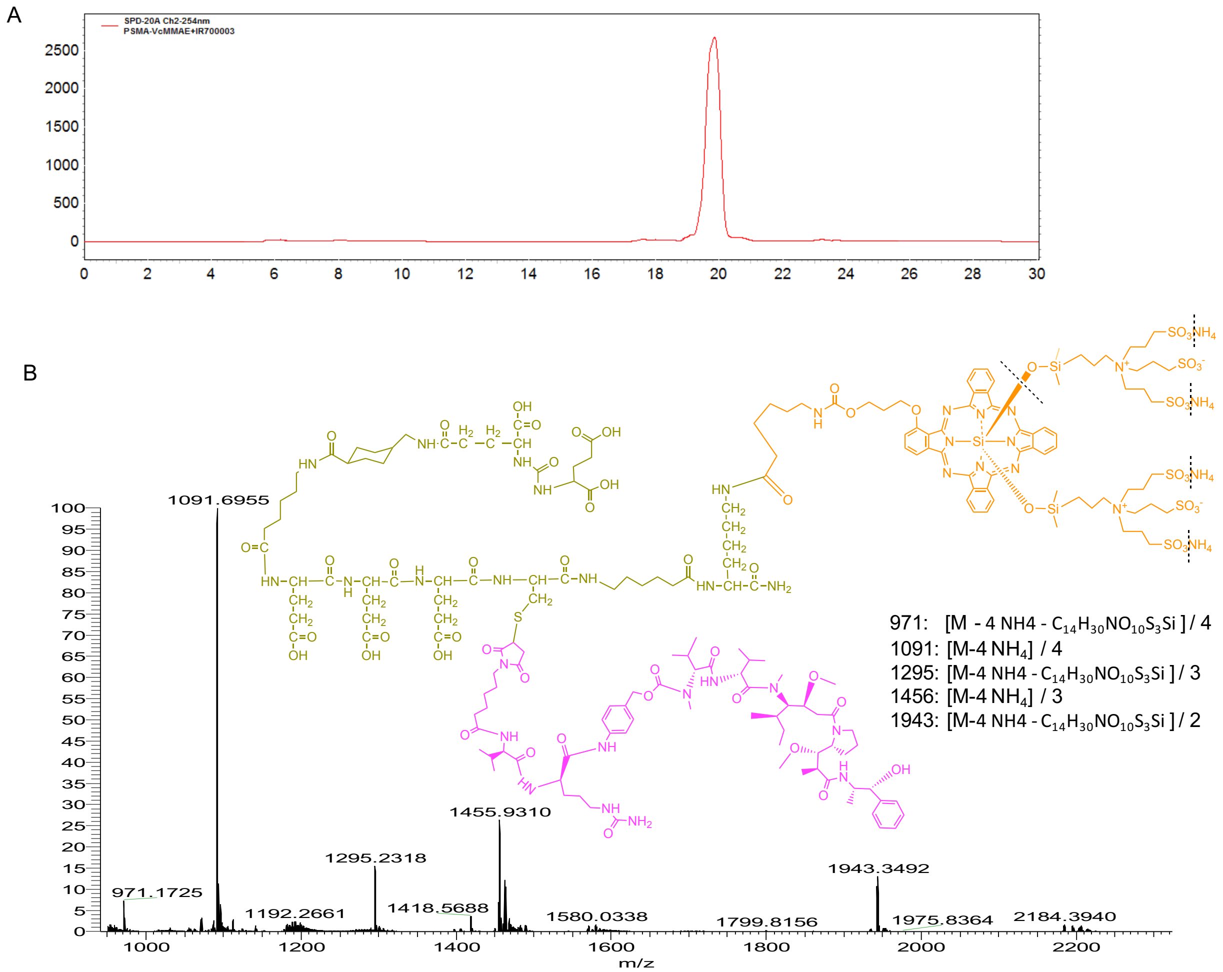

Figure S4. Characterization of PSMA-1-MMAE-IR700. (A) HPLC of purified PSMA-1-MMAE-IR700. HPLC gradient: 10-90% acetonitrile against 25 mM triethylammonium acetate (TEAA, pH 7.5) over 30 min. Retention time at 19.9 min. (B) Mass spectrum of PSMA-1-MMAE-IR700. MS spectrum was performed in negative mode.

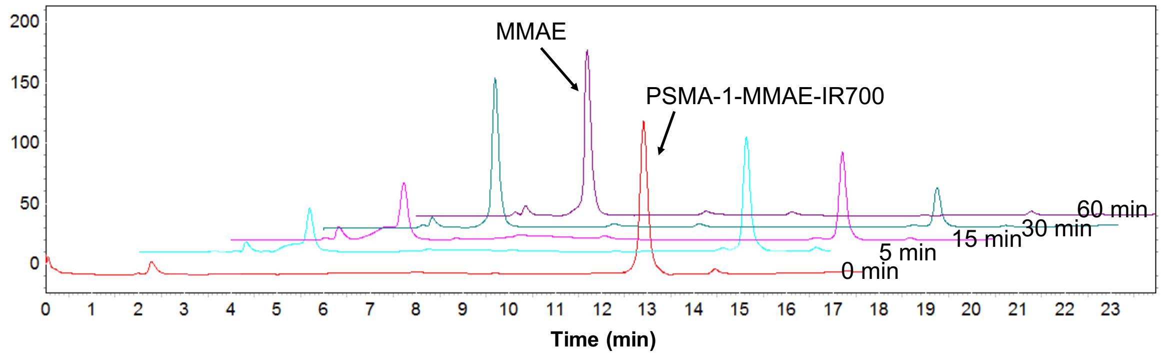

Figure S5. Chromatograms of PSMA-1-MMAE-IR700 degradation and release of MMAE when incubated with cathepsin over time. HPLC gradient: 40-90% acetonitrile against 25 mM triethylammonium acetate (TEAA, pH 7.5) over 30 min.

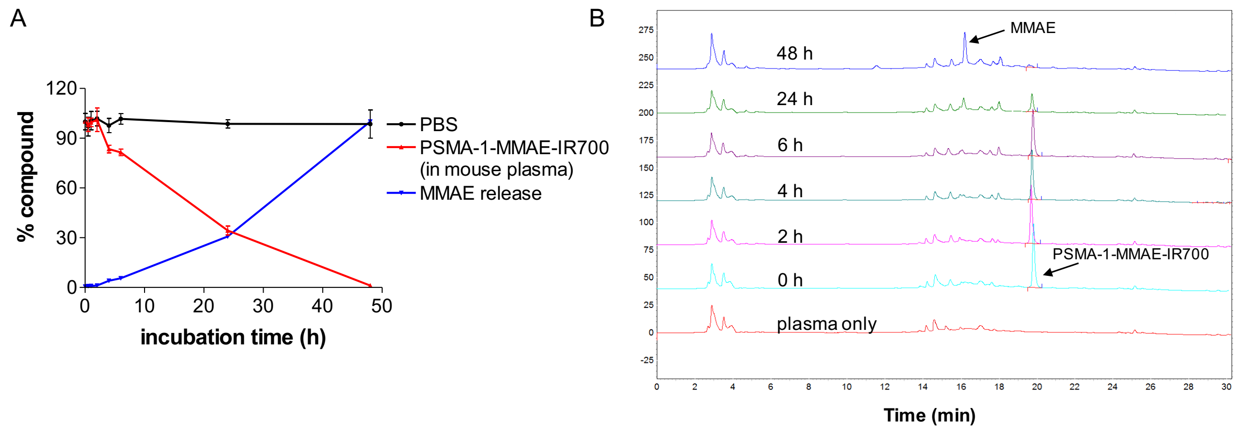

Figure S6. Stability of PSMA-1-MMAE-IR700. PSMA-1-MMAE-IR700 in PBS or mouse plasma was incubated at 37 ºC in the dark. Aliquotes (40 μL) were taken at different time points. To the aliquoted plasma, 40 μL of acetonitrile was added and the mixture was centrifuged to precipitate plasma protein. The supernatant was then analyzed by HPLC using the following gradient: 20% acetonitrile and 80% 25mM TEAA for 5 min, then 20-100% acetonitrile against 25 mM TEAA over 25 min. Studies were performed in triplicates. (A) Stability of PSMA-1-MMAE-IR700 in PBS and mouse plasma. Values are mean ± SD of triplicates. (B) HPLC profile of PSMA-1-MMAE-IR700 in mouse plasma.

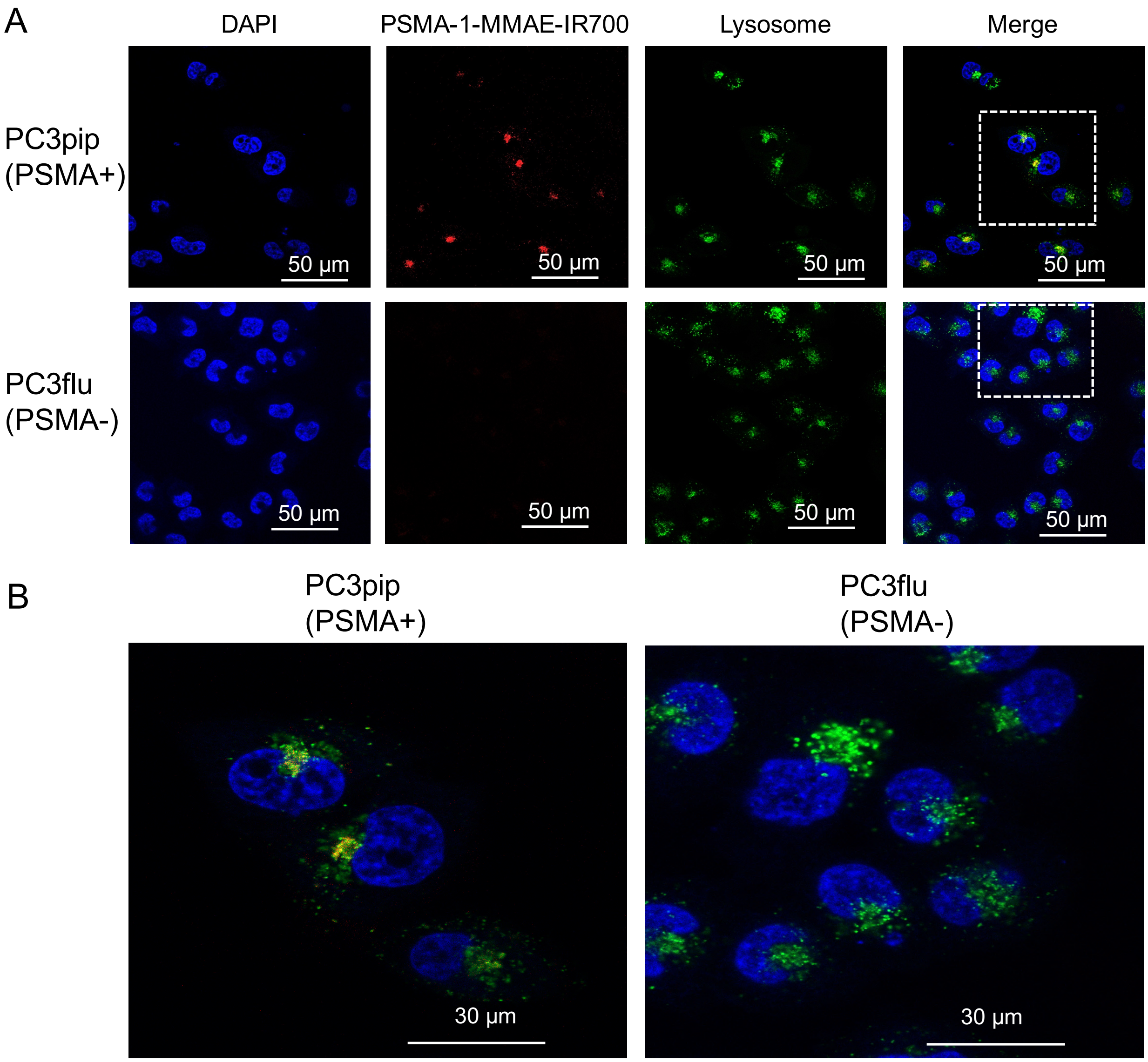

Figure S7. Confocal fluorescence images of uptake of PSMA-1-MMAE-IR700 in cells. (A) Cells were incubated with 50 nM of PSMA-1-MMAE-IR700 for 4 h. Nuclei were stained with DAPI and are false colored blue, lysosomes were detected by LysoOrange and are false colored green and PSMA-1-MMAE-IR700 signals are false colored red. Selective uptake was observed only in PC3pip cells and the drug was mainly located in lysosomes. Representative images are shown from three independent experiments. (B) Enlarged images of white squares in A.

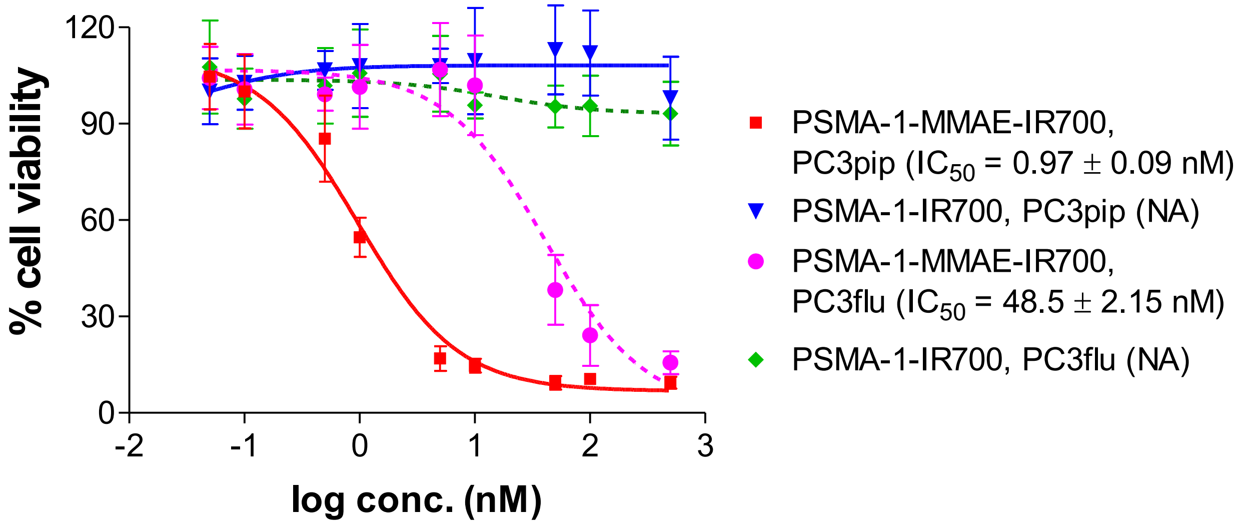

Figure S8. Dark cytotoxicity of PSMA-1-MMAE-IR700 with 72-hour incubation in the dark. NA means IC_{50} value is not available. Values are mean ± SD of six replicates.

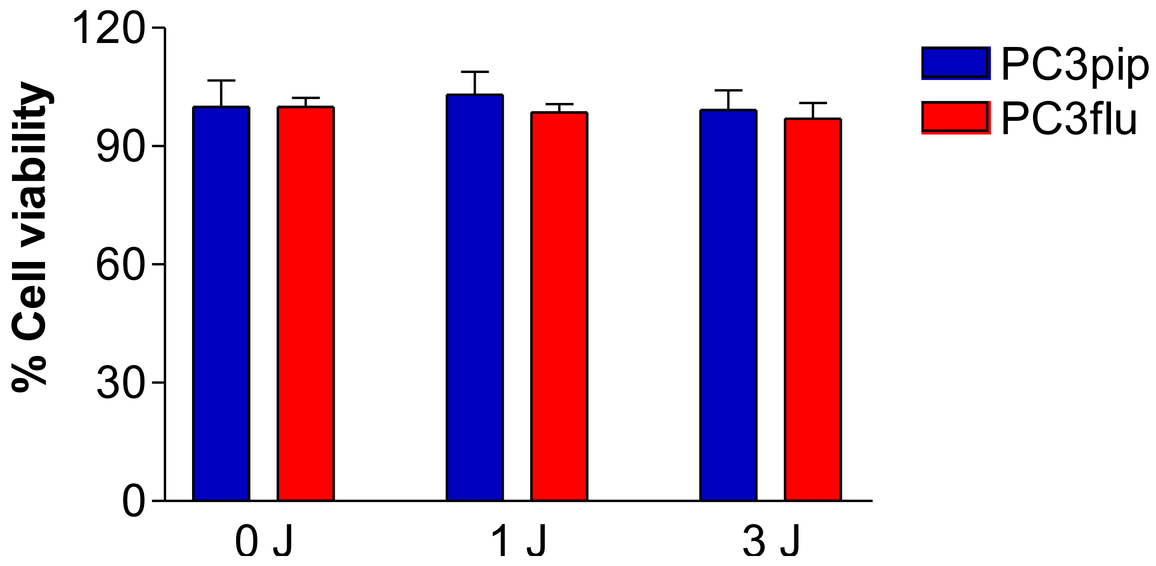

Figure S9. Light treatment of cells. PC3pip and PC3flu cells in RMPI 1640 media were treated with 690 nm light at 1 J/cm^2 and 3 J/cm^2. Light treatment didn't cause cells death. Values are mean ± SD of six replicates.

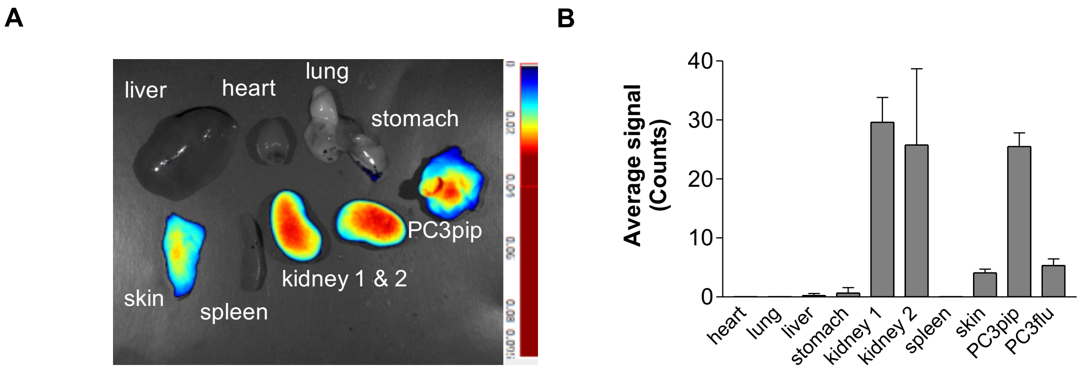

Figure S10. *Ex vivo* imaging of tissues from mice bearing orthotopic PC3pipGFP tumor. (A). *Ex vivo* fluorescence images of organs. Mice received PSMA-1-MMAE-IR700 and organs were taken out at 1 h post injection. Representative images are shown of n=3. (B) Quantification of fluorescent signal intensity in tissues. Values are mean±SD of 3 animals.

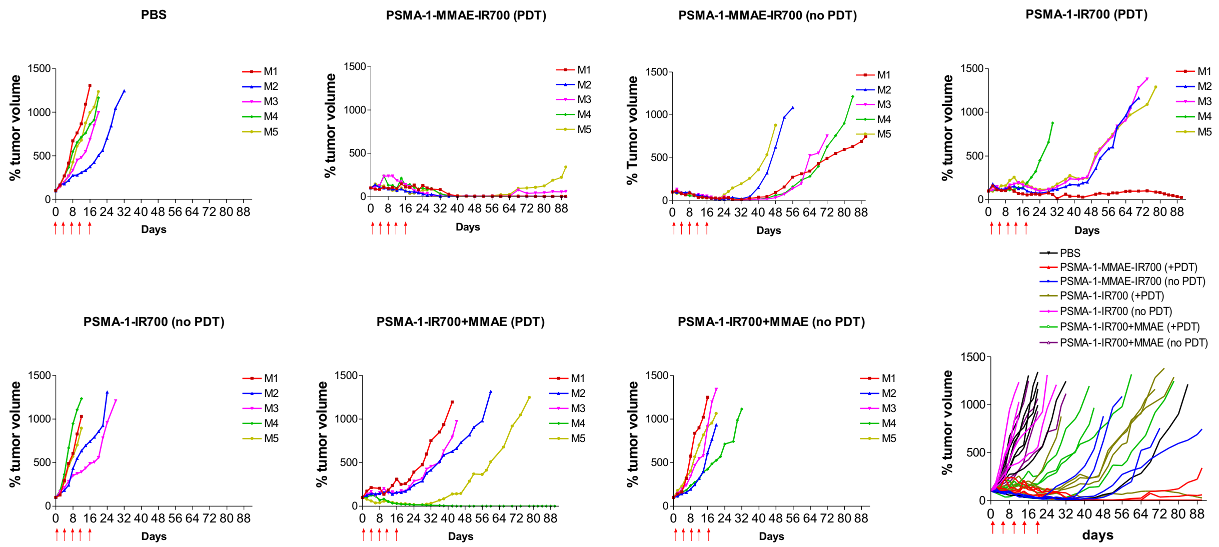

Figure S11. Tumor growth curves of different treatment groups. Treatment was scheduled every 4 days with a total of five doses as indicated by the red arrows. Each group had 5 mice. The last figure in the lower panel is the combination of tumor growth curves from all treatment groups.

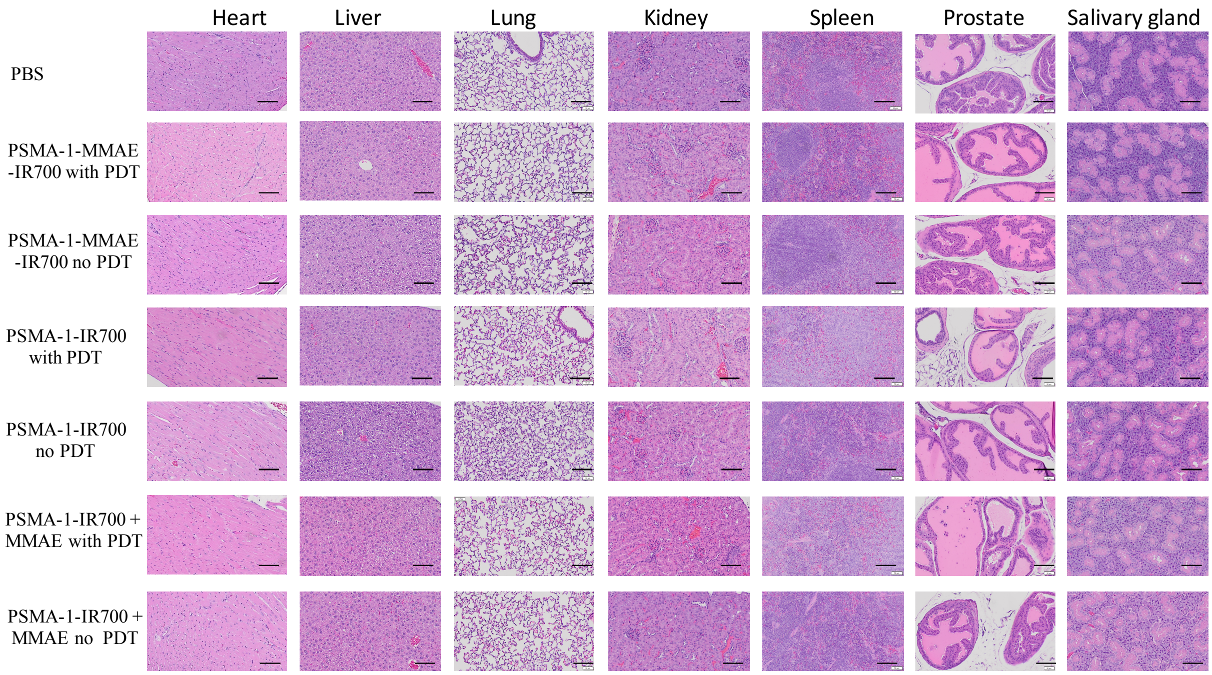

Figure S12. Histological examination of major organs after treatment. Mice receive 100 nmol/kg of drugs through tail vein injection every 4 days with a total of 5 injections. For the groups treated with PDT, light irradiation was performed at 1 hour post inject. Mice were sacrificed on day 30 and organs excised for H&E staining. The pictures are representative images of 3 mice.

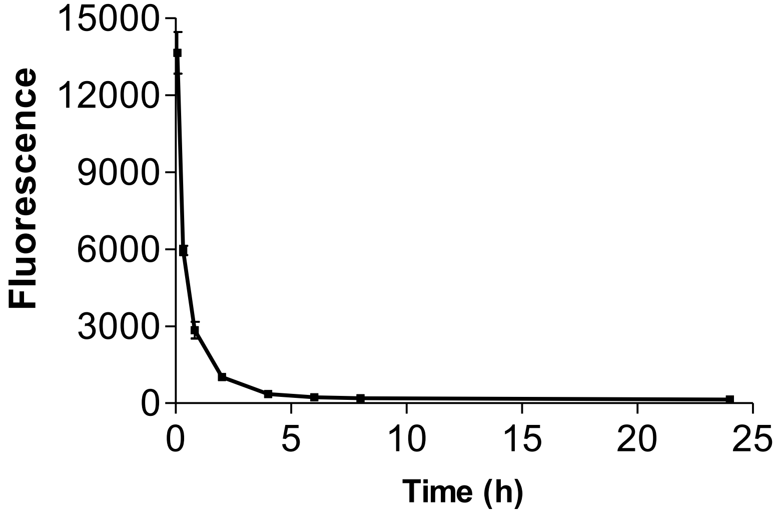

Figure S13. Pharmacokinetic studies of PSMA-1-MMAE-IR700 in mice. Mice received 100 nmol/kg of PSMA-1-MMAE-IR700 through tail vein injection. Blood was taken at 5 min, 20 min, 50 min, 1 h, 2 h, 4 h, 6 h, 8 h and 24 h. Fluorescence in the blood plasma was then measured (excitation 630 nm, emission at 690 nm). Values are mean±SD of 3 animals.

Table S1. Summary of P values of Kaplan-Meier Survival curves.

group	group	p-value
**PSMA-1-MMAE-IR700 (+ PDT)**	PSMA-1-MMAE-IR700 (no PDT)	0.0483
**PSMA-1-MMAE-IR700 (+ PDT)**	PSMA-1-IR700 (+ PDT)	0.0456
**PSMA-1-MMAE-IR700 (+ PDT)**	PSMA-1-IR700 (no PDT)	<.0001
**PSMA-1-MMAE-IR700 (+ PDT)**	PSMA-1-IR700+MMAE (+PDT)	0.0426
**PSMA-1-MMAE-IR700 (+ PDT)**	PSMA-1-IR700+MMAE (no PDT)	<.0001
**PSMA-1-MMAE-IR700 (+ PDT)**	ctl	<.0001
**PSMA-1-MMAE-IR700 (no PDT)**	PSMA-1-IR700 (+ PDT)	0.9736
**PSMA-1-MMAE-IR700 (no PDT)**	PSMA-1-IR700 (no PDT)	0.0265
**PSMA-1-MMAE-IR700 (no PDT)**	PSMA-1-IR700+MMAE (+PDT)	0.8754
**PSMA-1-MMAE-IR700 (no PDT)**	PSMA-1-IR700+MMAE (no PDT)	0.0400
**PSMA-1-MMAE-IR700 (no PDT)**	ctl	0.0475
**PSMA-1-IR700 (+ PDT)**	PSMA-1-IR700 (no PDT)	0.0288
**PSMA-1-IR700 (+ PDT)**	PSMA-1-IR700+MMAE (+PDT)	0.9015
**PSMA-1-IR700 (+ PDT)**	PSMA-1-IR700+MMAE (no PDT)	0.0432
**PSMA-1-IR700 (+ PDT)**	ctl	0.0490
**PSMA-1-IR700 (no PDT)**	PSMA-1-IR700+MMAE (+PDT)	0.0390
**PSMA-1-IR700 (no PDT)**	PSMA-1-IR700+MMAE (no PDT)	0.8678
**PSMA-1-IR700 (no PDT)**	ctl	0.7632
**PSMA-1-IR700+MMAE(+PDT)**	PSMA-1-IR700+MMAE (no PDT)	0.0479
**PSMA-1-IR700+MMAE(+PDT)**	ctl	0.0478
**PSMA-1-IR700+MMAE(no PDT)**	ctl	0.8928
